# scChIX-seq infers dynamic relationships between histone modifications in single cells

**DOI:** 10.1038/s41587-022-01560-3

**Published:** 2023-01-02

**Authors:** Jake Yeung, Maria Florescu, Peter Zeller, Buys Anton de Barbanson, Max D. Wellenstein, Alexander van Oudenaarden

**Affiliations:** 1 Oncode Institute, Hubrecht Institute-KNAW (Royal Netherlands Academy of Arts and Sciences) and University Medical Center Utrecht, Utrecht, the Netherlands; 2 Institute of Science and Technology Austria (ISTA), Klosterneuburg, Austria

**Keywords:** Histone post-translational modifications, Machine learning, Histone analysis

## Abstract

Regulation of chromatin states involves the dynamic interplay between different histone modifications to control gene expression. Recent advances have enabled mapping of histone marks in single cells, but most methods are constrained to profile only one histone mark per cell. Here, we present an integrated experimental and computational framework, scChIX-seq (single-cell chromatin immunocleavage and unmixing sequencing), to map several histone marks in single cells. scChIX-seq multiplexes two histone marks together in single cells, then computationally deconvolves the signal using training data from respective histone mark profiles. This framework learns the cell-type-specific correlation structure between histone marks, and therefore does not require a priori assumptions of their genomic distributions. Using scChIX-seq, we demonstrate multimodal analysis of histone marks in single cells across a range of mark combinations. Modeling dynamics of in vitro macrophage differentiation enables integrated analysis of chromatin velocity. Overall, scChIX-seq unlocks systematic interrogation of the interplay between histone modifications in single cells.

## Abstract (web summary)

Analysis of two histone marks in single cells reveals interplay between modifications.

## Main

Gene expression in animals relies on epigenetic marks such as histone modifications to regulate the accessibility and function of the genome in different cell types^1^. Large-scale efforts characterizing different histone modifications in a variety of cell populations commonly use chromatin immunoprecipitation followed by sequencing (ChIP–seq)^2–8^. Alternative strategies to ChIP–seq based on enzyme tethering (chromatin immunocleavage, ChIC) have reduced the background signal in profiling the epigenome^9^, and have enabled single-cell profiling of histone modifications^8,10–19^. Tethering strategies involve incubating cells with an antibody against a histone modification of interest, which then tethers either protein A-MNase^10,12,18,19^ or protein A-Tn5^11,13–17^ fusion protein to generate targeted DNA fragments in single cells. However, most experimental techniques to map single-cell histone modifications are limited to only one histone modification per single cell.

**Fig. 1:**
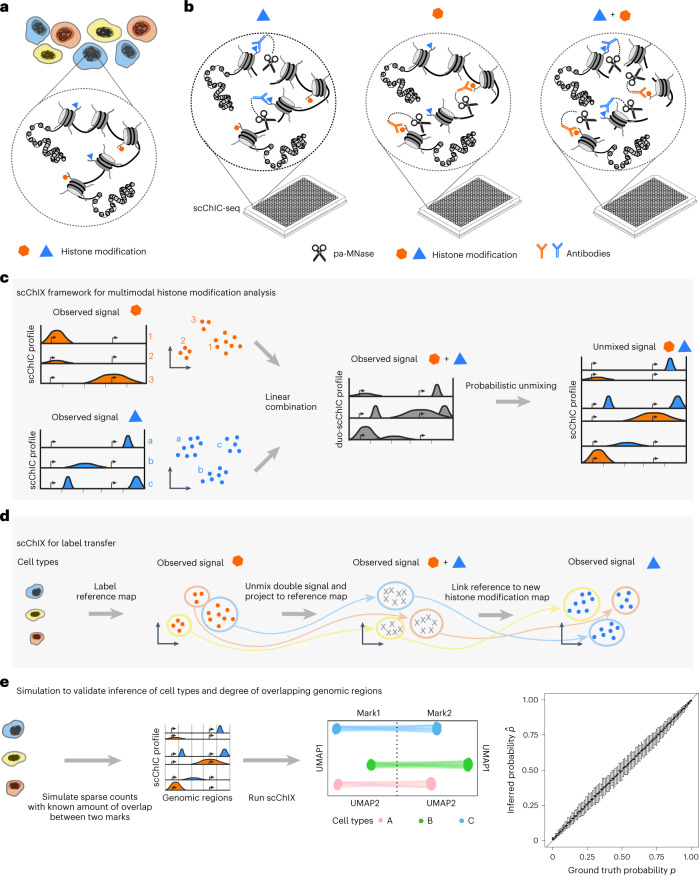
Overview of the scChIX-seq method. **a**, Chromatin regulation of different cell types (different colored cells) is regulated in part through several histone modifications (two histone modifications shown as an example). **b**, scChIX-seq uses three sortChIC antibody incubation conditions: two conditions each target a single histone modification (single-incubated) only and the third condition targets both histone modifications simultaneously (double-incubated). **c**, Schematic of scChIX-seq for deconvolving multiplexed histone modifications. The two single-incubated sortChIC datasets (one targeting an orange histone modification, the other a blue modification, each modification reveals three clusters) are training data to define the possible pairs of histone modification distributions that can be combined to generate a hypothetical double-incubated cell. For each observed double-incubated cell, we then assign the cell to the most probable pair of cell states, one from each histone modification. We then probabilistically assign each pA-MNase cut into their respective histone modification. Cartoons represent genome-wide distribution of histone modification signals in different modifications and cell types; *x* axes represent genomic distance, and vertical ticks are arbitrary distance markers. **d**, Label transfer allows joint analysis of two single-incubated sortChIC datasets targeting functionally distinct histone modifications. Information derived from one histone modification, such as cell types, histone mark levels and pseudotime, can be transferred to another histone modification using the double-incubated cells as a link. **e**, Simulation study shows that scChIX-seq can unbiasedly assign reads to each mark regardless of the amount of overlap there is between the two marks across the genome. *x* axis of cartoon genome-wide distributions (middle-left) is genomic distance. Right: ground truth probabilities versus inferred probabilities from scChIX. *p* is the expected fraction of double-incubated reads in a genomic locus that belongs to mark 1. $\hat{p}$ is the estimate of the probability; *n* = 101 simulation datapoints spread evenly between 0 and 1 inclusive. Error bars are 95% CI, centers are the mean.

We present an integrated experimental and computational framework for multiplexing histone modifications in single cells. To profile two histone modifications in single cells (Fig. 1a), we first generate three genome-wide sortChIC^18^ datasets: two datasets by incubating cells with one of the two histone modification antibodies separately (single-incubated; Fig. 1b), and the third by incubating cells with both histone modification antibodies together (double-incubated; Fig. 1b). We then use our two single-incubated datasets as training data to generate the possible pairs of genome-wide histone modification profiles that, when added together, fit to a single-cell profile from the double-incubated dataset (Fig. 1c). For each double-incubated cell, we then deconvolve the multiplexed data by probabilistically assigning each fragment back to their respective histone modification.

scChIX-seq links single-cell maps of different histone modifications, revealing relationships between histone modifications in single cells. In these linked maps, information derived from one chromatin state, such as cell types, histone mark levels and pseudotimes, can transfer to another chromatin state (Fig. 1d), unlocking joint analysis of several histone modifications in single cells. We first validated scChIX-seq using simulation, purified blood cell types and whole bone marrow. We then applied scChIX-seq to two complex biological systems, one in mouse organogenesis to uncover orthogonal dynamics in H3K36me3 and H3K9me3, and the other in macrophage in vitro differentiation to reveal coordinated dynamics between H3K4me1 and H3K36me3.

## Results

### Benchmarking across histone modification relationships

**Extended Data Fig. 1:**
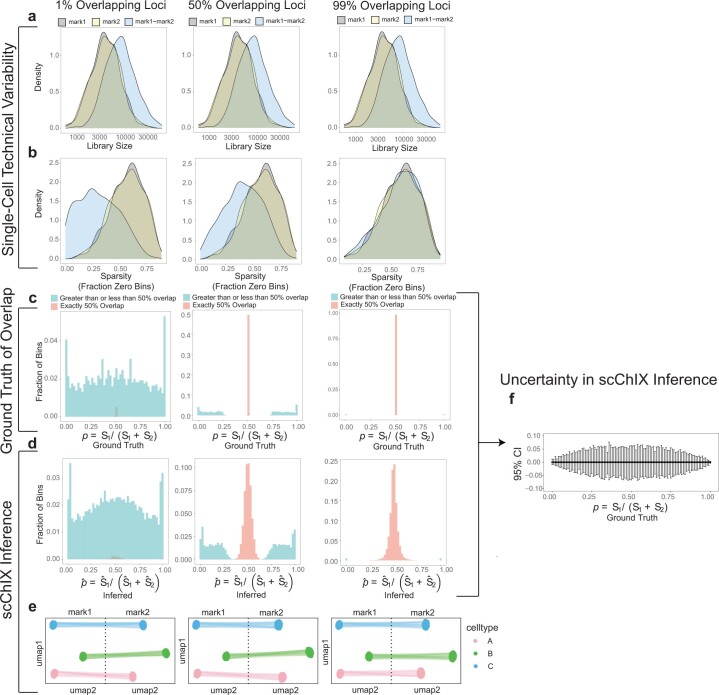
Benchmarking scChIX-seq across a range of overlapping patterns. Left column: simulation results in a mutually exclusive scenario (that is 1% of loci are overlapping). Middle column: results for an intermediate amount of overlap (that is 50% of loci are overlapping). Right column: results for highly correlated scenario (that is 99% of loci are overlapping). **(a)** Distribution of unique fragment cuts per cell in simulation. **(b)** Sparsity of the input matrix. Note that in the mutually exclusive scenario, the double-incubated marks is less sparse than single-incubated marks because loci with zero reads in one mark often have non-zero reads in another mark. **(c)** Distribution of the degree of overlap (defined as fraction of double-incubated signal belonging to mark1: $p=\frac{{S}_{1}}{{S}_{1}+{S}_{2}}$) for each locus genome-wide. **(d)** Estimated degree of overlap from scChIX-seq. **(e)** UMAP representation of the three cell types underlying simulation. UMAPs from the two marks are linked by double-incubated cells that are deconvolved by scChIX-seq. **(f)** Empirical 95% confidence interval across the range of $\hat{p}=\frac{{\hat{S}}_{1}}{{\hat{S}}_{1}+{\hat{S}}_{2}}$ (from 0 to 1). Range obtained by aggregating results from the three overlapping patterns. n=101 simulation datapoints spread evenly between 0 and 1 inclusive. Error bars are empirial 95% confidence intervals, centers are the mean.

To test whether scChIX-seq is accurate for histone modification patterns that are mutually exclusive as well as highly overlapping, we apply scChIX-seq to simulated single-cell data with known amounts of overlap to benchmark our method across different overlapping patterns between histone modifications. We simulate single-cell histone modification data by modifying simATAC^20^ to generate sparse count data from different overlapping patterns from the same cell (Fig. 1e and Extended Data Fig. 1a,b; Methods). Our simulations span three scenarios to cover varying degrees of overlapping patterns (Extended Data Fig. 1c). (1) Mutually exclusive scenario with only 1% of loci overlapping. (2) Intermediate scenario with 50% of loci overlapping. (3) Correlated scenario with 99% of loci overlapping. In these simulations, we provide a ground truth parameter *p* for each genomic locus and then estimate this parameter using our statistical framework to assess the uncertainty in our inferences. Here, *p* is the expected fraction of double-incubated reads in a locus that belongs to a reference histone modification (that is, *p* = 0.5 if locus is exactly overlapping, *p* = 1 or 0 if locus is exactly mutually exclusive). Applying scChIX-seq to each scenario, we find that the distribution of our estimates $\hat{p}$ across all loci are comparable with the ground truth distribution of *p* (Extended Data Fig. 1c,d). Furthermore, scChIX-seq accurately recovers the different cell types underlying the simulated data, and links the two histone modification landscapes into a joint uniform manifold approximation and projection (UMAP) (Extended Data Fig. 1e). Summarizing the three scenarios, scChIX-seq can estimate *p* accurately for all degrees of overlap, with confidence intervals (CI) better than $\hat{p}\pm 0.05$ (Fig. 1e (right) and Extended Data Fig. 1f). Our simulation study confirms that scChIX-seq is accurate in inferring several histone modifications in single cells in both mutually exclusive as well as overlapping histone modification patterns.

### Validating with ground truth data from purified cell types

**Extended Data Fig. 2:**
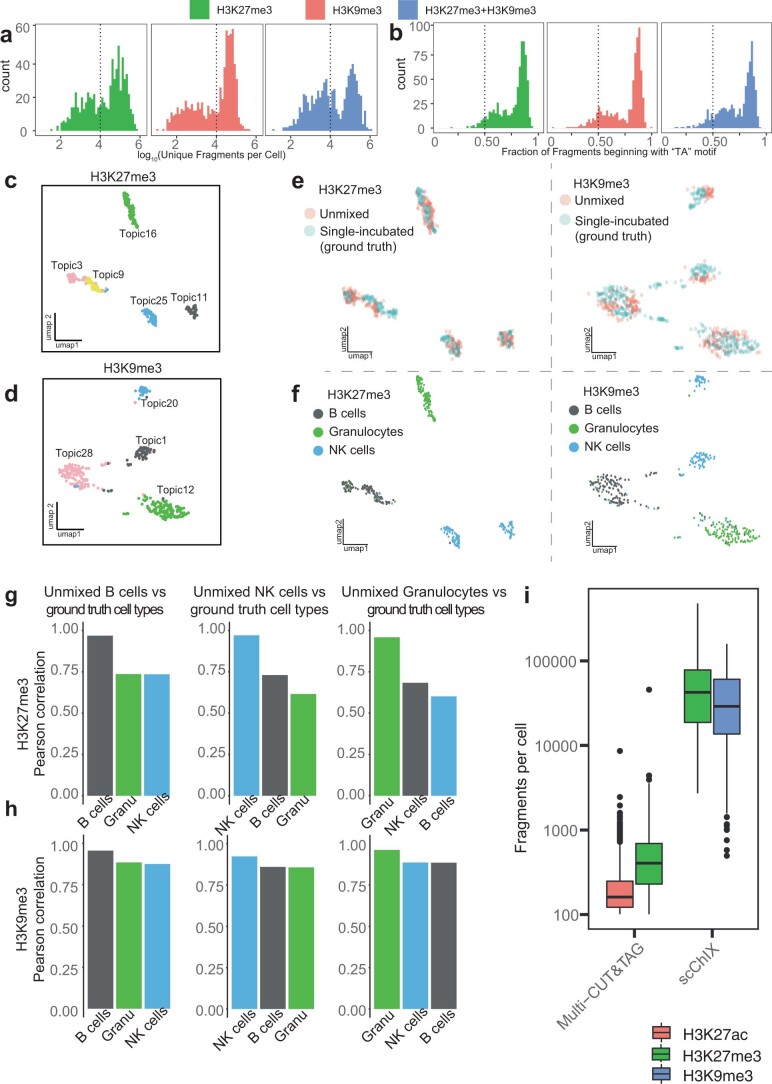
scChIX-seq accurately deconvolves double-incubated signal into their respective histone modifications. **(a)** Histogram of unique fragment cuts per cell. **(b)** Histogram of fraction of unique fragments starting with a "TA" motif. **(c, d)** UMAP of latent Dirichlet allocation (LDA) embedding using k=30 topics for H3K27me3 (c) and H3K9me3 (d). **(e, f)** UMAP representation of H3K27me3 (left) and H3K9me3 (right) data colored by unmixed or single-incubated cells (e) or ground truth cell type labels defined by FACS (f). **(g, h)** Genome-wide Pearson correlation between deconvolved H3K27me3 (g) and H3K9me3 (h) signal versus ground truth sortChIC purified by FACS. Shared genomic regions were calculated by using 1 kb bins across the genome. **(i)** Comparison of fragments per cell obtained from Multi-CUT&TAG versus scChIX-seq. Multi-CUT&TAG data came from a mixture of embryonic and trophoblast stem cells *in vitro*, while scChIX-seq came from sorted bone marrow cells *in vivo*. n=1806 cells for Multi-CUT&TAG, n=290 for scChIX-seq.

To validate our method experimentally, we generate a ground truth sortChIC dataset by purifying three known cell types from mouse bone marrow: B cells, granulocytes and natural killer (NK) cells, using fluorescence-activated cell sorting (FACS) and applying scChIX-seq (Methods). Of note, the sortChIC method is designed to integrate FACS with histone modification mapping^18^, so we can enrich for a cell type and map histone modifications in one workflow. We split bone marrow cells into three technical batches: one batch incubated with anti-H3K27me3 antibody alone (single-incubated), one with anti-H3K9me3 alone (single-incubated) and the third with both anti-H3K27me3 and anti-H3K9me3 antibodies together (double-incubated, H3K27me3+H3K9me3). We then sorted cells into 384-well plates, each plate containing all three cell types, and generate targeted cut fragments (Extended Data Fig. 2a,b). We chose H3K27me3 and H3K9me3 because they have been shown to have a mutually exclusive relationship^21^, allowing us to verify whether we can infer the correct cell type as well as the generally mutually exclusive relationship. Of note, although H3K27me3 and H3K9me3 are known to be nonoverlapping, it is unclear how this relationship precisely changes to make cell-type-specific patterns at different loci, and therefore modeling the two relationships is still needed to accurately infer the two chromatin profiles in individual cells.

From the double-incubated data alone, we would not know which cut fragments correspond to H3K27me3 and which to H3K9me3, but would observe only a superposition of the two profiles. We therefore used the single-incubated sortChIC data to train a statistical model of how cells from the same cell type combine their H3K27me3 and H3K9me3 profiles to generate double-incubated cut fragments. This model was then used to deconvolve the single-cell multiplexed signal into their respective histone modifications (Methods).

To learn an interpretable latent space for H3K27me3 and H3K9me3, we applied latent Dirichlet allocation (LDA)^22,23^ to the single-incubated H3K27me3 and H3K9me3 datasets, which factorizes count matrices based on a multinomial model (Methods). (Extended Data Fig. 2c,d). LDA learns cell-type-specific vectors of probabilities. These parameters model the probability that a cut fragment would fall into a specific genomic region. These probabilities can therefore be interpreted as genome-wide histone modification distributions that depend on cell type, and each cell generates a high-dimensional sparse count vector with *n* total fragments by drawing *n* independent trials from these multinomial distributions.

Demultiplexing the double-incubated data involves two steps. First, we used the training data to infer which genome-wide H3K27me3 distribution was added to which H3K9me3 distribution to generate a linear combination of two distributions (H3K27me3+H3K9me3). Second, we probabilistically assigned each double-incubated cut fragment to either H3K27me3 or H3K9me3, given that we know the underlying linear combination of the two profiles.

**Fig. 2:**
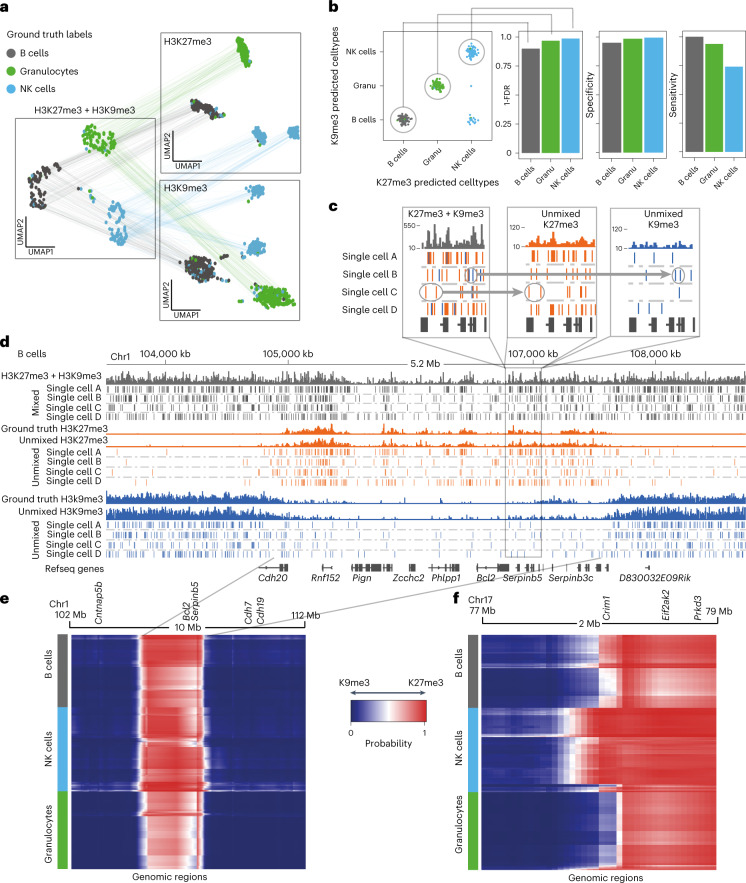
scChIX-seq accurately deconvolves multiplexed histone modifications in single cells. **a**, UMAP representation of the H3K27me3 (*n* = 367) and H3K9me3 (*n* = 376) histone modification space derived from the two single-incubated datasets (right two panels), and the H3K27me3+H3K9me3 space (left panel, *n* = 290) derived from the double-incubated data. Cells are colored by their ground truth cell-type labels. The cells in the H3K27me3- and H3K9me3-only space have unmixed double-incubated cells whose deconvolved signal has been projected onto their respective UMAPs. Lines connecting across datasets connect where each double-incubated cell is located in each of the three histone modification space. **b**, Matrix summarizing the cluster pair that scChIX-seq selected for each double-incubated cell. Cells along the diagonal are predicted to be B cells, granulocytes and NK cells, respectively. Cells in the off-diagonal are false negatives. Barplots summarizing FDR, sensitivity and specificity of assigning each cell type (right). **c**, Zoom-in coverage plot and single-cell cut fragments in B cells of mixed (H3K27me3+H3K9me3, gray bars), unmixed (H3K27me3 and H3K9me3, orange and blue bars). Positions of cut fragments are shown for four single cells (single cells A, B, C and D) for H3K27me3+H3K9me3 signal (gray ticks) as well as their unmixed outputs (orange and blue ticks). Circled reads and arrow highlight examples of cut fragments being assigned to either H3K27me3 (orange) or H3K9me3 (blue). **d**, Zoom-out of the *Serpinb5* locus. Cut fragments from H3K27me3+H3K9me3 are colored based on whether they have been assigned to H3K27me3 (orange) or H3K9me3 (blue). Ground truth coverage are single-incubated sortChIC data targeting H3K27me3 (orange) and H3K9me3 (blue). **e**, Heatmap of probabilities *p* of assigning reads to H3K27me3 (*p* = 1, red) or H3K9me3 (*p* = 0, blue) around the *Bcl2* locus. Rows are single cells (ordered by predicted cell type), columns are genomic regions (50 kb bins). Transitions between H3K9me3- and H3K27me3-marked chromatin states are independent of cell type. **f**, Same as **e** but at the *Crim1* locus, where transitions from H3K9me3 to H3K27me3 (blue to red) are cell-type specific.

The deconvolved H3K27me3+H3K9me3 data generated two sets of cuts for each cell: one set coming from H3K27me3 and the other from H3K9me3. We projected the two sets of cuts onto the H3K27me3 or H3K9me3 latent space (learned from LDA), respectively (Fig. 2a). Since each deconvolved cell has a set of cuts in H3K27me3 and H3K9me3 simultaneously, we can link the UMAPs together, creating a joint chromatin regulation space (Fig. 2a).

The double- and single-incubated cells in the H3K27me3 and H3K9me3 UMAPs intermingle, suggesting that the model accurately assigns cut fragments to their respective histone modification (Extended Data Fig. 2e,f). Comparing the H3K27me3 deconvolved pseudobulk signal with our ground truth single-incubated pseudobulk shows high correlation for the expected cell type, and lower for the other two cell types (Extended Data Fig. 2g). The H3K9me3 deconvolved pseudobulk signal also shows highest correlation with the expected cell type, with lower correlation from other cell types (Extended Data Fig. 2h). Finally, we compared the fragments per cell obtained from scChIX-seq versus multi-CUT&TAG^24^, and found that scChIX-seq achieves higher sensitivity than multi-CUT&TAG (Extended Data Fig. 2i). Overall, our ground truth dataset demonstrates that scChIX-seq is accurate and sensitive in assigning cut fragments to their respective histone modification.

To quantify the accuracy of scChIX-seq in selecting the correct H3K27me3-H3K9me3 cluster pair to mix together, we color each cell by its ground truth label and plot its inferred H3K27me3-H3K9me3 pair on a two-dimensional (2D) grid (Fig. 2b, left). The false discovery rates (FDRs) of scChIX-seq predicting B cells, granulocytes or NK cells are 10%, 3% and 1%, respectively (Fig. 2b, right). Similarly, scChIX-seq has high specificity and sensitivity in inferring the correct cluster pairs (Fig. 2b, right).

**Extended Data Fig. 3:**
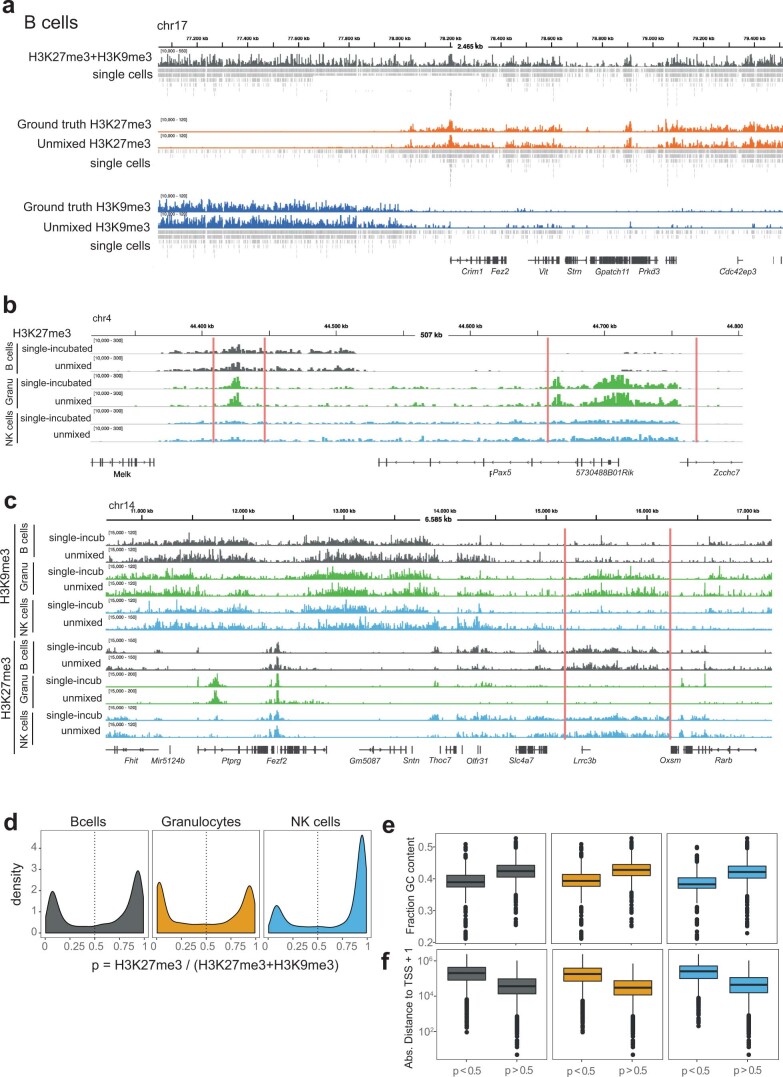
Coverage tracks of deconvolved cells and genome statistics. **(a)** Coverage tracks for B cells visualizing the H3K27me3+H3K9me3, deconvolved H3K27me3 or H3K9me3, and ground truth H3K27me3 or H3K9me3 histone modification levels for three different genomic regions. Double-incubated signal in grey, H3K27me3 single, and unmixed signal in orange, and H3K9me3 single and unmixed signal in blue. Under each coverage track are cut fragments of single cells. Each row of the single cells track are cuts from an individual cell. Shown are a subset of cells, which were chosen for their high number of cuts in the region. Rows are in decreasing order of total number of cuts. (**b)** H3K27me3 coverage tracks showing the region around *Pax5* for the ground truth H3K27me3 pseudobulk signal from single-incubated cells and for the deconvolved H3K27me3 pseudobulk signal from double-incubated cells for three cell types: B cells (grey), granulocytes (green), and NK cells (blue). (**c)** H3K9me3 (top) and H3K27me3 (bottom) coverage tracks showing the region around *Auts2* for ground truth (single-incubated) and for the unmixed (unmixed) for B cells (grey), granulocytes (green) and NK cells (blue), respectively. **d** Distribution of assignment probability estimates in the genome for the three cell types. Vertical dotted lines represent cutoffs to define H3K9me3-specific (that is *p* < 0.5) or H3K27me3-specific regions (that is *p*≥0.5). **e** Boxplot distributions of GC content in H3K27me3-marked and H3K9me3-marked regions. **f** Boxplot distributions of distance to TSS in the two classes of regions. Distances are measured from the center of the 50 kb locus to the nearest TSS. Number of bins in each boxplot: n=9962 for B cells *p* < 0.5, n=15877 for B cells *p*≥0.5, n=12483 for granulocytes *p* < 0.5, n=13345 for granulocytes *p*≥0.5, n=7337 for NK cells *p* < 0.5, n=18491 for NK cells *p*≥0.5. Boxplots show 25th percentile, median and 75th percentile, with the whiskers spanning 97% of the data.

Next, scChIX-seq assigns each double-incubated cut fragment to either H3K27me3 or H3K9me3 (Fig. 2c; Methods). The deconvolved B cell repressive landscapes correspond with their respective ground truth, exemplified in the *Bcl2* (Fig. 2d) and *Crim1* (Extended Data Fig. 3a) locus. We also find cell-type-specific signal in H3K27me3 (Extended Data Fig. 3b) and H3K9me3 signal (Extended Data Fig. 3c).

Our model infers *p*, the expected fraction of double-incubated fragments at a locus that belongs to H3K27me3. That is, *p* = 0 if all fragments belong to H3K9me3 and *p* = 1 if they all belong to H3K27me3. Plotting these probabilities across all loci reveals a bimodal distribution with peaks near 0 and 1 (Extended Data Fig. 3d). Classifying these loci as H3K9me3-specific (*P* < 0.5) or H3K27me3-specific (*P* ≥ 0.5), we compare the GC content and distance to transcription start site (TSS) of the two classes of loci (Extended Data Fig. 3e,f). We find H3K9me3-specific regions to have lower GC content and increased distance from TSSs compared with H3K27me3-specific regions. Of note, we observe this difference across all three cell types, suggesting that GC-poor and gene-poor regions of the genome is a general feature of H3K9me3-specific regions^21^.

Summarizing these probabilities in single cells along the genome as a heatmap, the *Bcl2* locus reveals the mutual exclusive relationship between H3K27me3 and H3K9me3, where the chromatin state is predominantly H3K9me3, then switches to H3K27me3, and then switches back to H3K9me3 (Fig. 2e). For *Bcl2*, these transitions occur at the same location independent of the cell type. However, we also find that these transitions can be cell-type specific, as exemplified by the *Crim1* locus (Fig. 2f), where the H3K27me3 region extends further upstream of *Crim1* in NK cells compared with B cells and granulocytes. Our ground truth experiment demonstrates that scChIX-seq can accurately map two histone modifications in single cells, and the inferred probabilities can be biologically interpreted as relationships between the two histone modifications in single cells.

### scChIX-seq reveals H3K4me1/H3K27me3 relationships in bone marrow

**Fig. 3:**
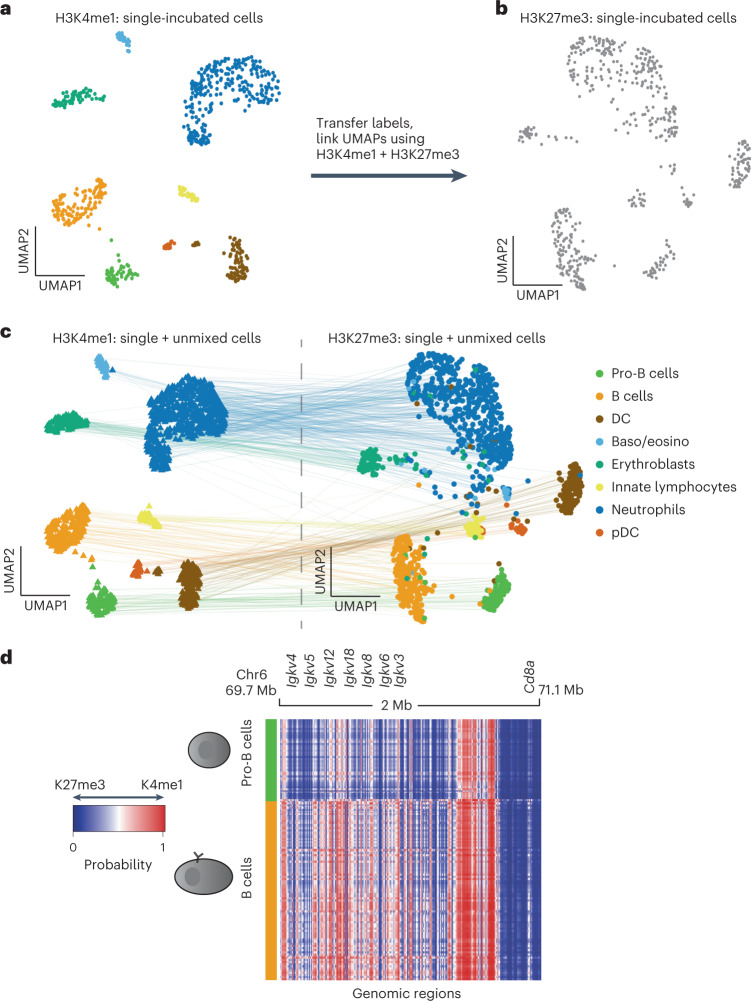
scChIX-seq enables joint analysis of distinct histone modifications in single cells. **a**, UMAP of sortChIC signal of H3K4me1 in bone marrow (*n* = 639 cells). Clusters are colored by cell type. Latent space calculated using LDA with 50 kb bins. **b**, UMAP of sortChIC signal of H3K27me3 in whole bone marrow (*n* = 517 cells). Cell types in H3K27me3 are inferred by transferring labels from H3K4me1. **c**, H3K4me1 and H3K27me3 UMAPs linked together by deconvolved double-incubated cells (*n* = 1,711 cells). H3K4me1 and H3K27me3 portions of the double-incubated cells are projected onto their respective UMAPs. Lines connect where the active signal and the corresponding repressive signal are located for each double-incubated cell. DC, dendritic cells; pDC, plasmacytoid dendritic cells. **d**, Heatmap showing probability of assigning a read in a region to either H3K27me3 or H3K4me1 at 5 kb resolution. Heatmap shows the *Igk* locus for pro-B versus B cells. Rows are single cells, columns are 5 kb genomic regions. Blue represents regions where cut fragments are probably coming from H3K27me3, while red represents regions where cut fragments are probably coming from H3K4me1.

**Extended Data Fig. 4:**
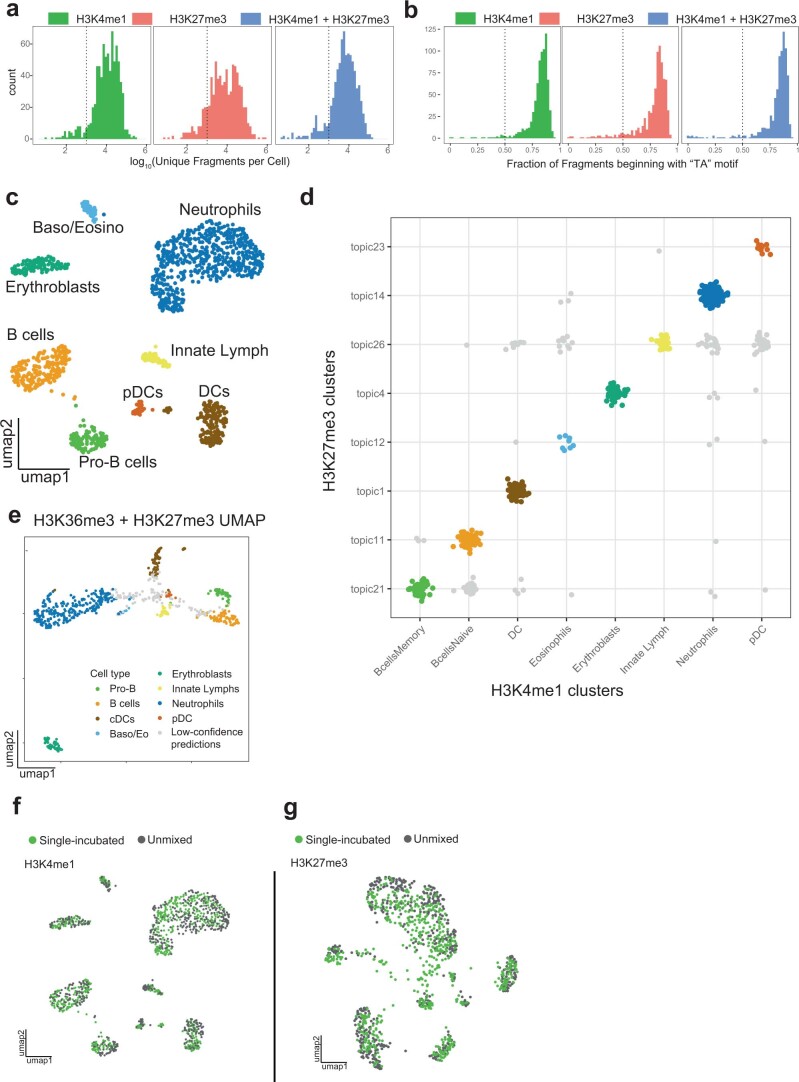
Inferring cluster pairs from H3K4me1+H3K27me3 transfers cell type labels. **(a)** Histogram of unique fragment cuts per cell. **(b)** Histogram of fraction of unique fragments starting with a "TA" motif. **(c)** UMAP of H3K4me1 sortChIC data, cells colored by cell type. **(d)** Assignment plot showing individual H3K4me1+H3K27me3 cells (represented as dots) assigned to a pair of topics (x-axis labels are H3K4me1 clusters, named by their associated cell type, while y-axis are H3K27me3 clusters). Cells along the diagonal are high-confidence predictions that match a H3K4me1 cluster with a H3K27me3 topics, and are colored by the H3K4me1-derived cell type labels. **(e)** UMAP of H3K4me1+H3K27me3 sortChIC. Cells are colored by their cell type inferred from cluster pairs. Low-confidence predictions are colored in grey. **(f, g)** UMAP representation of H3K4me1 (f) and H3K27me3 (g). Cells are colored by whether the epigenome was generated by single-incubation or by unmixing by scChIX-seq.

We next apply scChIX-seq to integrate active (H3K4me1) and repressive (H3K27me3) chromatin states in a complex mixture of cells by sampling mouse bone marrow (Extended Data Fig. 4a,b). We use scChIX-seq to transfer labels and link UMAPs between active and repressive histone modifications (Fig. 3a,b) to perform a joint analysis of the two marks.

To define cell types from the H3K4me1 sortChIC data, we ranked the top 150 genes associated with different clusters from sortChIC and used a publicly available scRNA-seq dataset to compare mRNA abundances of cluster-specific genes across different blood cell types^25^ (Extended Data Fig. 4c). scChIX-seq takes each H3K4me1+H3K27me3 cell and infers the most probable cluster pair (one from H3K4me1, the other from H3K27me3), which systematically transfers cell-type labels defined from H3K4me1 onto the H3K27me3 data (Extended Data Fig. 4d). We find that a small minority of double-incubated cells have low-confidence cluster pair predictions. Plotting the cluster pairs onto the H3K4me1+H3K27me3 UMAP confirms that the single-cell assignment produces precise clusters where neighboring cells are probably assigned to the same pair. Low-confidence predictions arise from cells that border between clusters (Extended Data Fig. 4e), which we remove from further analysis. Overall, scChIX-seq allows systematic transfer of cell-type labels from one histone modification to another.

**Extended Data Fig. 5:**
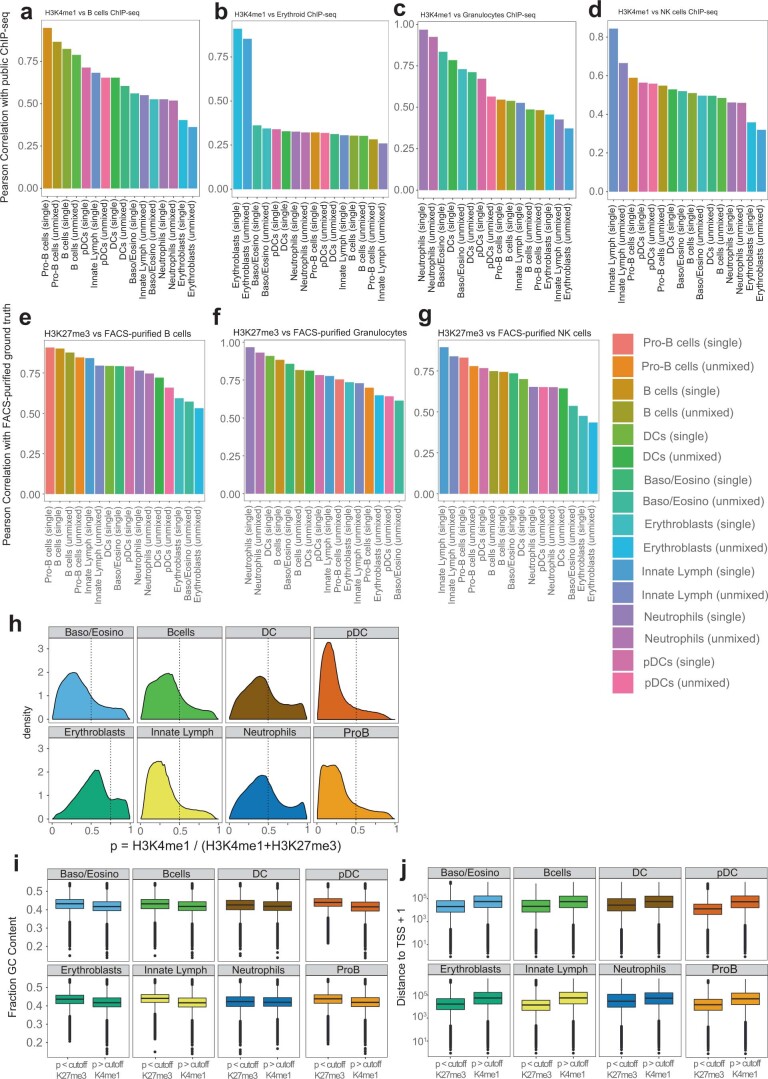
Histone modification signal of deconvolved cell types correlates with public H3K4me1 ChIP-seq and H3K27me3 sortChIC ground truth data. **(a-d)** Pearson correlation between publicly available H3K4me1 ChIP-seq^5^ data of purified B cells (a), erythroid (b), granulocytes (c), or NK cells (d) versus H3K4me1 profiles of different cell types derived from scChIX-seq. Single: pseudobulk profiles generated by single incubation, unmixed: pseudobulk profiles deconvolved by scChIX-seq. **(e-g)** Pearson correlation between H3K27me3 sortChIC from FACS-purified B cells (e), granuloytes (f), NK cells (g) versus H3K27me3 sortChIC derived from pseudobulks of whole bone marrow without FACS purification. Single: pseudobulk profiles generated by single incubation, unmixed: pseudobulk profiles deconvolved by scChIX-seq. **(h)** Distribution of assignment probability estimates *p* in the genome for the three cell types. Vertical dotted lines represent cutoffs for *p* to define H3K27me3-specific and H3K4me1-specific regions. *p* is the expected fraction of reads that belong to H3K4me1 in a specific genomic locus. **(i)** Boxplot distributions of GC content for the two classes of regions. **(j)** Boxplot distributions of distance to TSS in the two classes of regions. Distances are measured from the center of the 5 kb locus to the nearest TSS. Boxplots show 25th percentile, median and 75th percentile, with the whiskers spanning 97% of the data.

We next deconvolve the double-incubated cells into their respective histone modification. The UMAPs from H3K4me1 and H3K27me3 show that single-incubated and deconvolved single cells intermingle, suggesting that deconvolution does not produce batch effects (Extended Data Fig. 4f,g). The deconvolved single cells provide anchors to systematically link one histone modification with another (Fig. 3c). To validate the predicted cell types in both the single and deconvolved datasets, we compared with data from cell types purified by FACS. For H3K4me1 clusters, we compared with publicly available ChIP–seq^5^. Pearson correlation between ChIP–seq of B cells, erythroids, granulocytes and NK cells versus sortChIC from single- and double-incubated cells is highest for the predicted cell type (Extended Data Fig. 5a–d). Although single-incubated cells have higher correlation with ChIP–seq reference data than deconvolved cells for the matched cell type, the deconvolved cells of the matched cell type consistently had higher correlation with ChIP–seq than unmatched cell types. For H3K27me3 clusters, we used our ground truth sortChIC data purified from FACS. Pearson correlation of sortChIC signal between FACS-sorted B cells, granulocytes and NK cells versus pseudobulks derived from whole bone marrow is highest for the predicted cell type (Extended Data Fig. 5e–g).

Classifying these loci as H3K27me3-specific or H3K4me1-specific using a cluster-specific cutoff for *p* (Extended Data Fig. 5h), we again compare the GC content and distance to TSS of the two classes of loci. We find that H3K4me1-marked regions tend to be closer to TSSs compared with H3K27me3 (Extended Data Fig. 5i), and that GC content is higher in H3K27me3-specific compared with H3K4me1-specific regions (Extended Data Fig. 5j). The increase in GC content for H3K27me3-marked regions is consistent with previous studies showing that GC-rich elements in transcriptionally inactive regions can recruit PRC2 (ref. ^26^).

**Extended Data Fig. 6:**
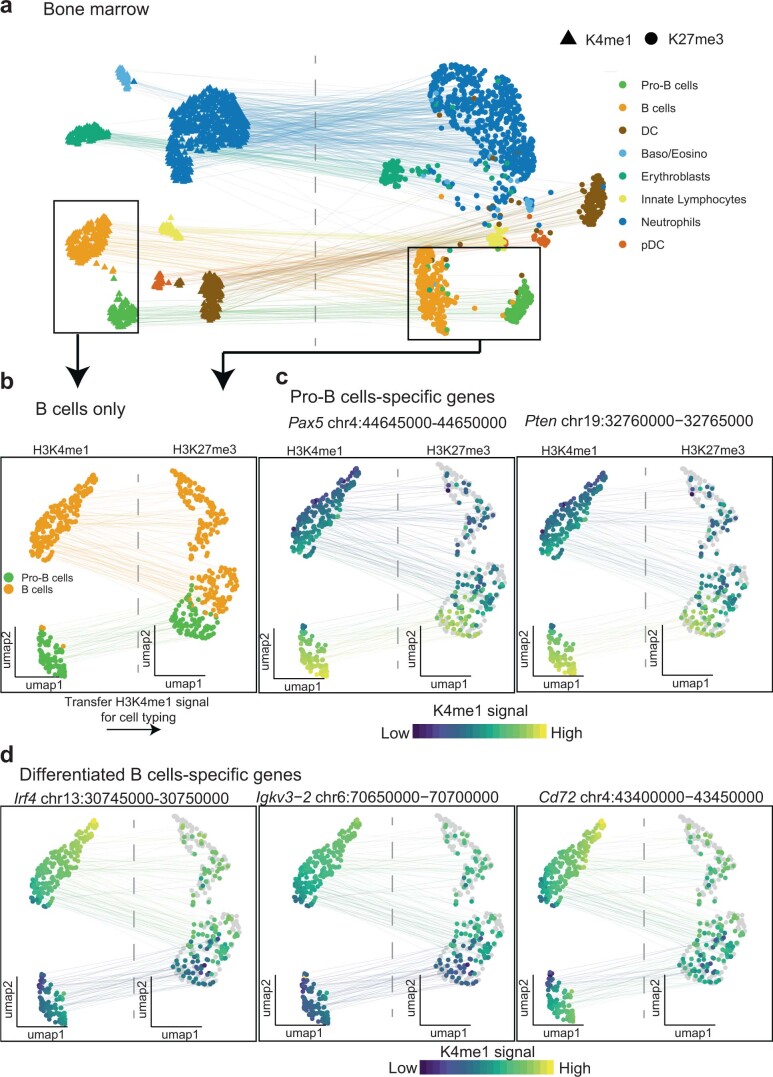
Re-clustering on B cells reveals heterogeneity within B cells. **(a)** UMAP visualization of H3K4me1 and H3K27me3 (single signal and unmixed signal), colored by cell types derived from H3K4me1 and transferred to H3K27me3. Black rectangle indicates the B cell population used to re-cluster in (b,c,d). **(b)** UMAP of pro-B and B cells only. **(c,d)** Projection of H3K4me1 signal of marker genes for pro-B (c) or for differentiated B cells (d). H3K4me1 signal is measured in all cells of the H3K4me1 UMAP (that is both single- and double-incubated have H3K4me1 signal in the H3K4me1 UMAP). Double- (colored) but not single-incubated (grey) cells have H3K4me1 signal in the H3K27me3 UMAP.

We use the joint landscape to reveal active and repressive histone modification dynamics within cell types. To find differences in chromatin regulation between pro-B cells versus B cells, we select only pro-B or B cells and recluster the cells in both H3K4me1 and H3K27me3 separately (Extended Data Fig. 6a,b). With multimodal data, we can transfer cell-type-specific H3K4me1 signal onto the H3K27me3 UMAP to distinguish pro-B and B cells with more confidence. Using pro-B cell-specific genes, *Pax5* (ref. ^27^) and *Pten*^28^, we project the H3K4me1 signal at loci overlapping these genes onto both H3K4me1 and H3K27me3 landscapes, confirming a subset of pro-B cells within the B cell population (Extended Data Fig. 6c). Similarly, we use marker genes associated with more differentiated B cells, such as *Irf4* (ref. ^27^), *Igkv3-2* locus^29^ and *Cd72* (ref. ^30^) to confirm a more differentiated B cell population (Extended Data Fig. 6d). Plotting the heatmap of H3K4me1-H3K27me3 assignment probabilities at the *IgK* locus reveals that the chromatin state is repressed in pro-B cells but becomes activated in B cells (Fig. 3d), consistent with the progressive activation of the chromatin state during B cell development^29^.

**Extended Data Fig. 7:**
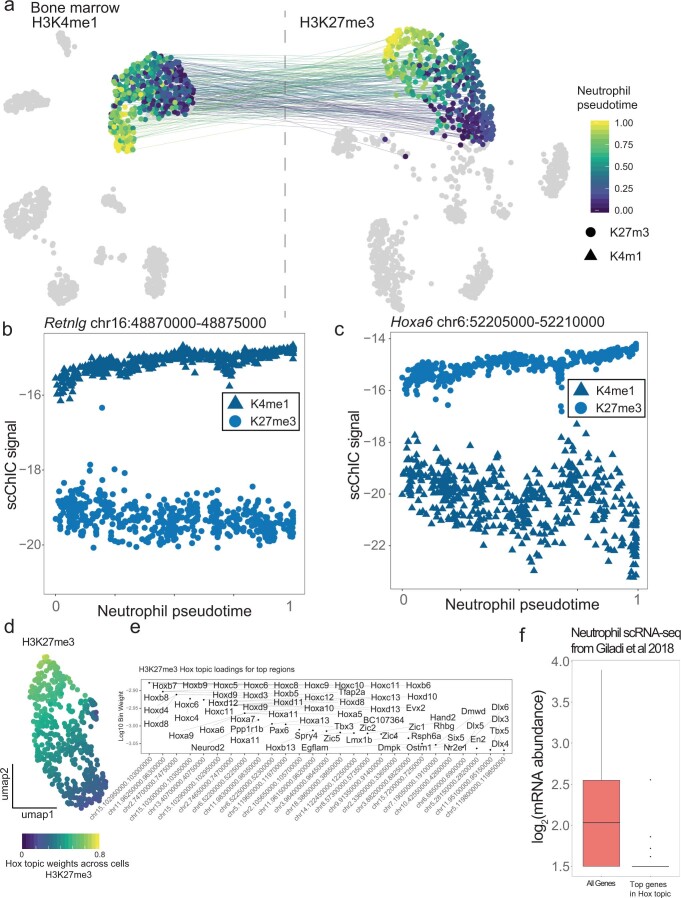
H3K4me1 and H3K27me3 signal during neutrophil maturation. **(a)** UMAP visualization of H3K4me1 and H3K27me3, lines join H3K4me1 and H3K27me3 UMAPs of double-incubated neutrophils. Heterogeneity within neutrophils are colored as neutrophil pseudotime. **(b)** H3K4me1 and H3K27me3 modification levels at the *Retnlg* (a mature neutrophil marker gene) locus along neutrophil pseudotime. **(c)** H3K4me1 and H3K27me3 modification levels at the *Hoxa* along neutrophil pseudotime. **(d)** UMAP of H3K27me3 signal across single cells colored by weights of a topic containing high H3K27me3 levels at many *Hox* and developmental gene loci (*Hox* topic). **(e)** Topic weights of the top 150 genes associated with loci in the *Hox* topic for H3K27me3. **(f)** Neutrophil mRNA abundance of genes in the *Hox* topic compared to other genes derived from publicly available scRNA-seq data^25^. Number of genes per boxplot: n=17986 for All Genes, n=127 for genes in the Hox topic. Boxplots show 25th percentile, median and 75th percentile, with the whiskers spanning 97% of the data.

Next, we recluster neutrophils to analyze differences in chromatin regulation along pseudotime (Extended Data Fig. 7a). Reclustering neutrophils in H3K27me3 reveals a shared pseudotime trajectory that varies smoothly between neutrophils in both the H3K27me3 and H3K4me1 landscapes. H3K4me1 levels at the *Retnlg* locus—a marker gene for mature neutrophils^31^—increases along pseudotime, while H3K27me3 levels decreases (Extended Data Fig. 7b). The H3K27me3 gene loadings associated with pseudotime consists of a module of *Hox* and other developmental genes (Extended Data Fig. 7c–e). Of note, these genes have low levels of mRNA abundances in neutrophils (Extended Data Fig. 7f), suggesting that this module is transcriptionally silent. At a locus overlapping the *Hoxa* locus, we find that H3K27me3 was highly marked while H3K4me1 was lowly marked across all neutrophils. Along pseudotime, H3K27me3 increases further, while H3K4me1 decreases further (Extended Data Fig. 7c). Our pseudotime analysis suggests that dynamics in histone modifications can occur even in regions associated with low-expressed genes.

### H3K36me3/H3K9me3 relationships during mouse organogenesis

**Fig. 4:**
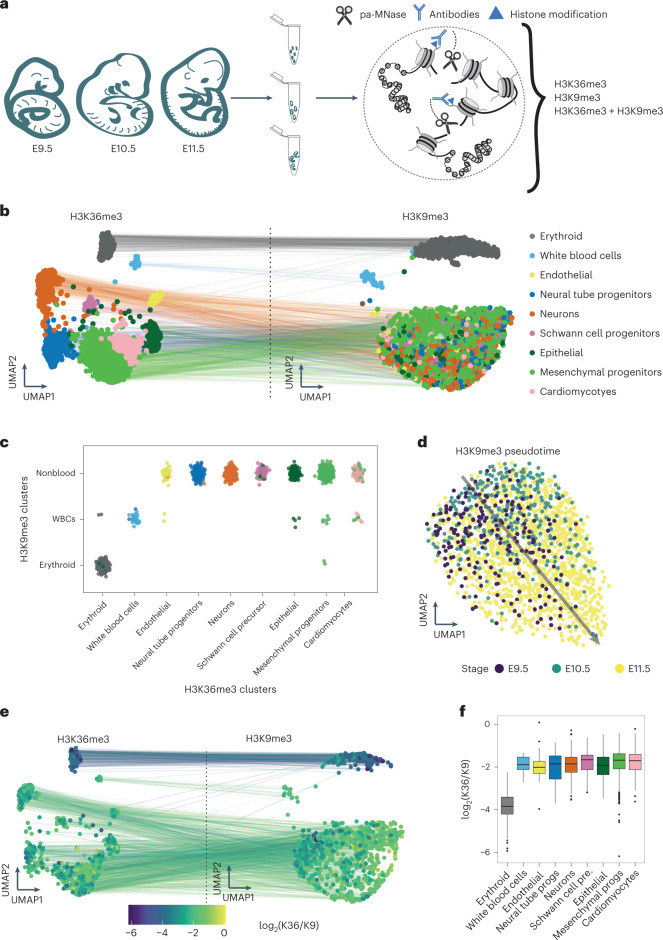
Applying scChIX-seq to mouse organogenesis reveals shared heterchromatin landscapes and cell-type-specific differences in H3K36me3:H3K9me3 ratios. **a**, Schematic of mouse organogenesis experiment to study H3K36me3 and H3K9me3 in single cells. **b**, Joint UMAP of mouse organogenesis after deconvolution from scChIX-seq (*n* = 2,911 H3K36me3 cells, *n* = 2,166 H3K9me3 cells). **c**, Assignment of several H3K36me3 cell types to one H3K9me3 cluster. The H3K36me3 (columns) and H3K9me3 (rows) label for each double-incubated cells (*n* = 1,197 cells) are plotted onto a matrix to H3K36me3 cell types to H3K9me3 clusters. Cells are colored by their cell-type label as in **b**. **d**, Subclustering of nonblood cells for H3K9me3, represented as a UMAP. Arrow denotes a pseudotime axis. Pseudotime defined as the first PC of the 2D UMAP. **e**, Joint UMAP of deconvolved double-incubated cells (*n* = 1,197 cells), colored by the log ratio of number of H3K36me3 cuts versus number of H3K9me3 cuts. **f**, Boxplot of H3K36me3:H3K9me3 ratio across cell types. Number of double-incubated cells for each cell type: *n* = 163 erythroid, *n* = 17 white blood cells, *n* = 24 endothelial, *n* = 136 neural tube progenitors, *n* = 197 neurons, *n* = 46 Schwann cell precursors, *n* = 73 epithelial, *n* = 458 mesenchymal progenitors and *n* = 83 cardiomyocytes. Boxplots show 25th percentile, median and 75th percentile, with the whiskers spanning 97% of the data.

**Extended Data Fig. 8:**
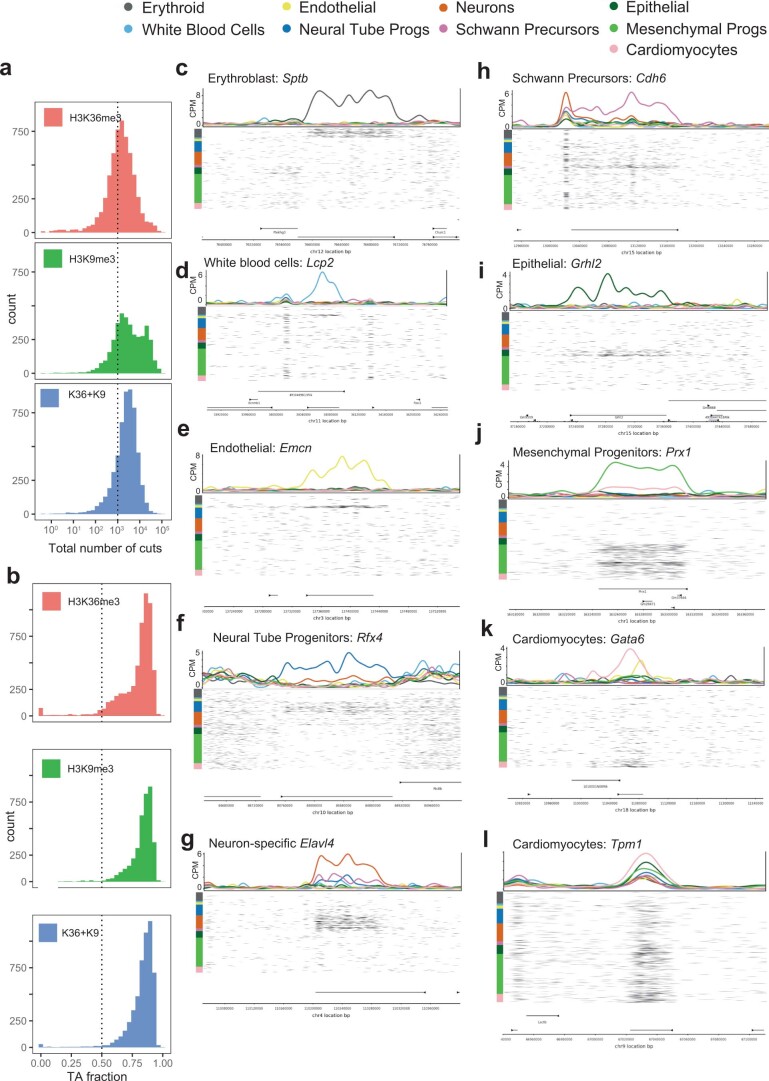
Cell typing mouse organogenesis dataset using H3K36me3 using marker genes. **(a)** Histogram of unique fragment cuts per cell. **(b)** Histogram of fraction of unique fragments starting with a "TA" motif. **(c-l)** Genome browser plots of cell type-specific H3K36me3 loci showing pseudobulk CPM signals (colored lines, top) and cut locations of individual cells (bottom, black marks). Cells are ordered by cell type (color-coded on the left).

To demonstrate the method in more complex biological scenarios, we applied scChIX-seq during mouse organogenesis (E9.5 to E11.5) to study H3K36me3 and H3K9me3 dynamics at single-cell resolution (Fig. 4a and Extended Data Fig. 8a,b). We took the top 250 cluster-specific bins from the H3K36me3 data to identify cell types (Methods). These loci associate with gene bodies of cell-type-specific genes. For example, we find H3K36me3 signal around genes enriched in specific cell types, such as erythroids (*Sptb*)^32^, white blood cells (*Lcp2* (ref. ^33^), endothelial cells (*Emcn*)^34^, neural tube (*Rfx4*)^35^, neurons (*Elavl4*)^36^, Schwann precursors (*Cdh6*)^37^, epithelial cells (*Grhl2*)^38^, mesenchymal progenitors (*Prx1*)^39^ and cardiomyocytes (*Gata6*, *Tpm1*)^40,41^ (Extended Data Fig. 8c–l).

**Extended Data Fig. 9:**
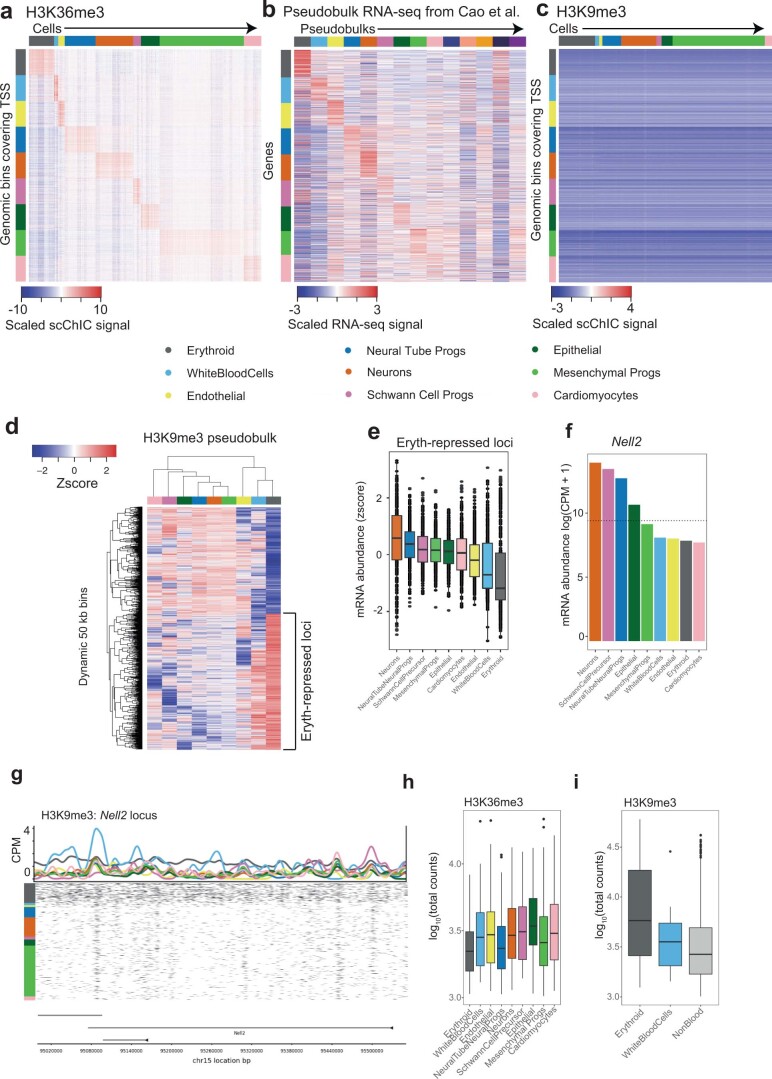
H3K9me3-specific regions across cell types. **(a)** Heatmap of H3K36me3 signal for the top 250 H3K36me3-specific loci (rows) across cell types (columns). **(b)** Heatmap of mRNA abundances for the genes associated with the H3K36me3-specific loci in (a) across pseudobulks. Data processed from publicly available scRNA-seq data from Cao et al.^42^. **(c)** Heatmap of H3K9me3 signal for the same top 250 H3K36me3-specific loci as in (a). The H3K36me3 and H3K9me3 heatmaps are mean-centered and scaled using a common mean and standard deviation calculated across both marks. **(d)** Heatmap of H3K9me3 signal across pseudobulks at H3K9me3-variable loci. **(e)** Relative mRNA abundances^42^ at n=364 genes associated with erythroblast-repressed loci across nine cell types. **(f)** mRNA abundance of an erythroblast-repressed gene, *Nell2*, across pseudobulks. **(g)** Genome browser plot of around the *Nell2* locus, an erythroblast-specific region for H3K9me3. Top of plot is pseudobulk H3K9me3 CPM signals, below are cut locations of individual cells (black marks). Cells are ordered by cell type (color-coded as in heatmaps). **(h, i)** Total unique fragments across cell types for single-incubated cells for H3K36me3 (h) and H3K9me3 (i), showing that the variability of the number of cuts across cells can span orders of magnitude. Number of single-incubated H3K36me3 cells for each boxplot: n=154 erythroid, n=36 white blood cells, n=60 endothelial, n=250 neural tube progenitors, n=272 neurons, n=58 Schwann cell precursors, n=154 epithelial, n=570 mesenchymal progenitors, n=160 cardiomyocytes. For H3K9me3: n=207 erythroid, n=26 white blood cells, n=736 non-blood cell types. Boxplots in (e), (h), (i) show 25th percentile, median and 75th percentile, with the whiskers spanning 97% of the data.

To uncover whether distinct H3K36me3 cell types could share common H3K9me3 landscapes, we deconvolved the H3K36me3 + H3K9me3 cells and projected each cell to both landscapes (Fig. 4b). scChIX-seq reveals that erythroid and white blood cells have both distinct active chromatin and heterochromatin, but the other nonblood cell types show similar heterochromatin distribution. Assigning each double-incubated cell to a H3K36me3 and H3K9me3 cluster confirms that cells with distinct H3K36me3 can share the same H3K9me3 cluster (Fig. 4c). Of note, the variable genes that show cell-type-specific differences in both active chromatin and publicly available mRNA abundances^42^ (Extended Data Fig. 9a,b) have low signal across cell types in H3K9me3 (Extended Data Fig. 9c), suggesting that using conventional marker genes from RNA-seq would not reveal cell-type differences in H3K9me3.

Differential expression across the three H3K9me3 clusters reveals cluster-specific repressed loci (Extended Data Fig. 9d), with the largest effect coming from erythroid-specific regions. These erythroid-repressed regions are associated with decreased mRNA abundances (Extended Data Fig. 9e–g). Subsetting the data and running LDA on only nonblood cells in H3K9me3, we find that H3K9me3 varies over organogenesis stages (Fig. 4d), suggesting that heterochromatin differences are stronger across organogenesis stages than between cell types.

Because the double-incubated cells have cut fragments associated with both histone modifications, we hypothesized that the deconvolved data could precisely quantify the ratio between the two histone modifications, and how this ratio changes across cell types. Counting total reads from single-incubated data would lead to large cell-to-cell technical variability because counts per cell can span several orders of magnitude. However, comparing the counts of the two histone modification in the same cell could overcome this technical variability. We therefore asked whether the global ratio of H3K36me3 and H3K9me3 in individual cells varies. Plotting the ratio of H3K36me3 and H3K9me3 reveals that most cells have comparable ratios, but that erythroid cells have lower ratios than other cell types (Fig. 4e,f). This lower ratio is consistent with mass spectrometry studies showing a global decrease in H3K36me3 but no change in H3K9me3 during erythroid maturation^43^. Of note, inferring this global change without scChIX-seq, such as by counting total unique fragments from single-incubation data, is challenging due to the large variability in total counts across cells and the inability to distinguish cell types in certain H3K9me3 clusters (Extended Data Fig. 9h,i).

In sum, applying scChIX-seq to H3K36me3 and H3K9me3 during organogenesis reveals unique insights from multimodal analysis. The complex relationships between the two histone modifications as well as their global changes would not have been elucidated by analyzing single-incubated data alone.

### Mark-specific pseudotimes and chromatin velocity

**Fig. 5:**
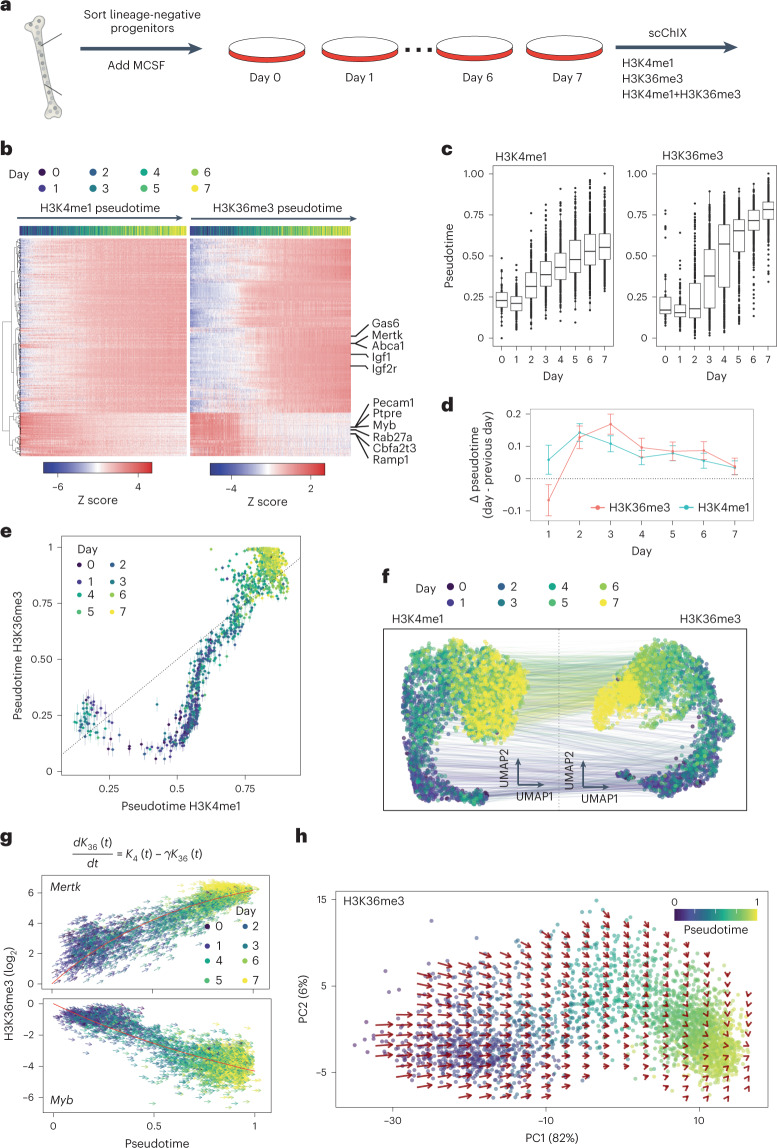
Applying scChIX-seq to two active marks reveals chromatin velocity during in vitro macrophage differentiation. **a**, Schematic of mouse macrophage in vitro differentiation timecourse experiment to study H3K4me1 and H3K36me3 in single cells. **b**, Heatmap of histone modification signal on the bodies of dynamic genes over pseudotime. Rows are gene bodies and columns are single-incubated cells ordered along pseudotime. Color labels of columns are days from which the cells were recovered during the timecourse. **c**, Boxplots of pseudotime estimates of single-incubated cells along the timecourse. Number of cells per day for H3K4me1: *n* = 58 day 0, *n* = 148 day 1, *n* = 249 day 2, *n* = 350 day 3, *n* = 369 day 4, *n* = 383 day 5, *n* = 488 day 6, *n* = 519 day 7. For H3K36me3: *n* = 42 day 0, *n* = 125 day 1, *n* = 176 day 2, *n* = 301 day 3, *n* = 384 day 4, *n* = 366 day 5, *n* = 522 day 6, *n* = 567 day 7. Boxplots show 25th percentile, median and 75th percentile, with the whiskers spanning 97% of the data. **d**, Estimate of the average difference of pseudotime from one day to the next. Error bars indicate 95% CI, calculated by a linear model of the pseudotime differences between days. Statistics derived from number of cells indicated in **c**. **e**, Estimates of two different pseudotimes from a single cell. Error bars are 95% CI of the estimates. Each point is a double-incubated cell. **f**, Joint UMAP of H3K4me1 and H3K36me3 from scChIX-seq, lines connect single cells with multimodal information. **g**, Chromatin velocity estimates of an upregulated gene (above) and a downregulated gene (below). Red curve is the exponential relaxation fit according to the solution of the first-order differentiation equation. **h**, High-dimensional chromatin velocities of dynamic genes projected onto PCs 1 and 2. Vector field estimated by smoothing across nearest neighbors of cells (Methods).

**Extended Data Fig. 10:**
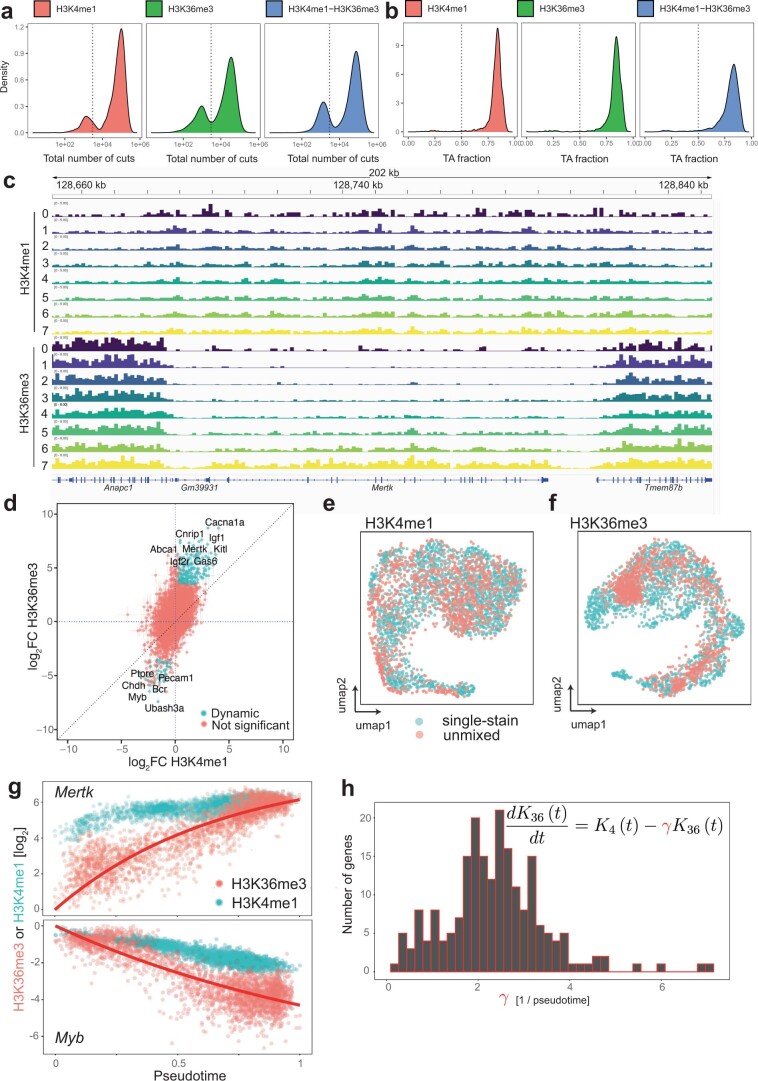
Distinct dynamics of H3K4me1 and H3K36me3 during macrophage in vitro differentiation. **(a)** Density plots of total number of cuts across cells for H3K4me1, H3K36me3, and H3K4me1+H3K36me3 labeled cells. **(b)** Density plots of fraction of cuts starting with a TA motif across cells for H3K4me1, H3K36me3, and H3K4me1+H3K36me3 labeled cells. **(c)** Genome-browser plot around gene body of *Mertk*, a macrophage-specific gene. Tracks are bigwigs from pseudobulks averaged across the time course. **(d)** Log2 fold change estimates along pseudotime on gene bodies in the genome. Colored dots are considered significant (log2 fold change in H3K36me3 > 3.5, zscore in both H3K36me3 and H3K4me1 > 2) and used for chromatin velocity estimates. **(e, f)** UMAP of H3K4me1 (e) and H3K36me3 (f) of single-incubated and deconvolved cells showing intermingling of the two types of cells. **(g)** Examples of H3K4me1 and H3K36me3 for an upregulated (above) and downregulated (below) gene along pseudotime. **(h)** Histogram of estimates of the rate constant *γ* for the 209 dynamic genes highlighted in (d).

Finally, we applied scChIX-seq to study the dynamic relationships between two active histone modifications, H3K4me1 and H3K36me3, over an in vitro differentiation timecourse. We sorted blood progenitors from mouse bone marrow, added macrophage colony-stimulating factor (MCSF) and collected cells over 7 days (Fig. 5a and Extended Data Fig. 10a,b; Methods). We incubated cells with either H3K4me1, H3K36me3 or both H3K4me1 and H3K36me3, then performed scChIX-seq.

Genome tracks of H3K4me1 and H3K36me3 signal for each day shows upregulation of macrophage-specific genes, such as *Mertk*^44^ (Extended Data Fig. 10c). Heatmap of H3K4me1 and H3K36me3 dynamics at gene bodies along pseudotime reveals that the two histone modifications up- and downregulate genes with different dynamics. H3K36me3 shows a gradual up- or downregulation of signal while H3K4me1 reaches a new steady state earlier along pseudotime (Fig. 5b). Summarizing log_2_ fold change of the two histone modifications genome-wide, we find that dynamics in H3K36me3 are often larger than in H3K4me1 (Extended Data Fig. 10d). Comparing pseudotime progression with day of sample collection shows that changes in H3K4me1 peak at day 2 and then increases progressively over the day while H3K36me3 dynamics peak around day 3 and 4 before relaxing towards steady state (Fig. 5c). The time of the largest change in H3K4me1 dynamics occurs 1 day before H3K36me3 (Fig. 5d), suggesting that global changes in H3K4me1 precede changes in H3K36me3. Summarizing at the genome-wide level, UMAPs of H3K4me1 and H3K36me3 of single-incubated cells show that both active marks move progressively towards a macrophage state during the timecourse (Fig. 5e).

Using continuous pseudotime of H3K4me1 and H3K36me3 as our training data (Methods), for both H3K4me1 and H3K36me3 we infer where along pseudotime each double-incubated cell came from. Plotting the inferred pseudotimes of each mark for each cell uncovers the dynamic relationships between the two marks (Fig. 5e). H3K4me1 pseudotime initially progresses while H3K36me3 remains relatively unchanged. As H3K4me1 pseudotime approaches 0.5, H3K36me3 then progresses rapidly towards 1. This sigmoidal-like relationship between H3K4me1 versus H3K36me3 pseudotime progression is consistent with H3K4me1 dynamics occurring before H3K36me3. Finally, we used this inferred pseudotime information to project the deconvolved cells onto the H3K4me1 and H3K36me3 UMAPs. Both UMAPs showed that the single-incubated and deconvolved cells intermingle with each other, suggesting that deconvolution was successful (Extended Data Fig. 10e,f). Using the deconvolved cells as anchors, we then linked the two histone modification maps together (Fig. 5f).

Since we observed that H3K4me1 dynamics occur before H3K36me3, we asked whether we could model the H3K36me3 dynamics as a first-order differential equation analogous to RNA velocity^45^ (Fig. 5g, top; Methods). Since our data come from a timecourse, we directly fitted the exponential curves for dynamic genes along pseudotime for H3K36me3 (Extended Data Fig. 10g), which avoids making steady-state assumptions and leverages information from both single-incubated and deconvolved cells across histone modifications. The distribution of inferred rate constants from the exponential fit show a median of approximately 2.3 per pseudotime (Extended Data Fig. 10h). These rate constants were then used to predict the H3K36me3 levels for each cell over small pseudotime steps (Δ*t* = 0.02; Fig. 5g). Finally, summarizing the predictions of dynamic genes, we projected the high-dimensional velocity vectors onto the first two principal components (PCs). From the chromatin velocity summary, we found that differentiation starts with large changes in H3K36me3 dynamics, and then relaxes towards the macrophage state.

In summary, we applied scChIX-seq to two active histone modifications to find dynamic relationships between activation states. We then model these dynamics to infer chromatin velocity during macrophage differentiation.

### Discussion

Here, we demonstrate that scChIX-seq can deconvolve multiplexed histone modifications, expanding the number of histone marks that can be profiled in single cells. Using simulations, purified cell types and whole bone marrow, we demonstrate that scChIX-seq can accurately map several histone marks. To show how scChIX-seq can reveal unique biological insights in more challenging systems, we applied scChIX-seq to study H3K36me3 and H3K9me3 dynamics during mouse organogenesis to reveal the joint transcriptional and heterochromatin relationships in single cells. scChIX-seq can identify complex cell-type relationships between histone modifications, such as when several cell types can share a similar heterochromatin landscape. Finally, we applied scChIX-seq to two active marks during macrophage in vitro differentiation to quantify the relationship between two correlating marks. Importantly, scChIX-seq is flexible in which histone modifications can be used. The correlation structure between modifications is inferred from the model and therefore does not require a priori assumptions of specific features of the two modifications. Thus, scChIX-seq complements a recent method that focuses on differences in fragment lengths between Pol2 serine-5 phosphate and H3K27me3 to assign reads to their respective mark^46^.

Recently, there have been new experimental innovations to CUT&TAG that modify the pA-Tn5 complex to map several histone modifications in single cells^24,47–49^. One drawback of Tn5-based approaches (for example, CUT&TAG) compared with MNase-based (for example, sortChIC and CUT&RUN) used in this study is that Tn5 can have biases to open chromatin^50^. Current CUT&TAG methods suppress this bias by using more stringent washing conditions^51^, but exceedingly high salt conditions reduce the sensitivity and could wash away weakly bound factors such as transcription factors^50,51^. On the flip side, MNase-based approaches involve more experimental effort than Tn5-based approaches, reducing the number of single cells that can be processed per round. Although we demonstrate our scChIX-seq method using an MNase-based approach (sortChIC), our computational and experimental framework can also be applied to Tn5-based strategies. Furthermore, our scChIX-seq method may have synergies with recent nanobody-based methods^47,48^. For example, using two nanobodies, each specific to a different species of immunoglobulin G, one can profile four histone modifications by generating two sets of scChIX-seq simultaneously: two antibodies raised from one species and the other two antibodies raised from the second species.

A limitation in scChIX-seq is that the maximum number of cuts at a specific base pair location is fundamentally limited by the copy number in that cell. Therefore, a nucleosome that has several modifications in their histone tails would still be cut only once. Currently, our binning strategy (5 kilobase (kb), 50 kb or gene bodies, depending on the biological question) and multinomial model assumes that there is no limit to the number of fragments that can be generated in one bin, which is an approximation that is valid when the bins are large and the number of cuts within the bins are small (for example, due to dropouts).

We demonstrate that scChIX-seq can reveal biological insights by multimodal analysis that would otherwise be obscured by analyzing each modality separately. Overall, scChIX-seq unlocks multimodal analysis in antibody-based chromatin profiling and enables joint analysis of distinct histone modifications in single cells.

## Methods

### Animal experiments

All mice used in this study were Cast-EiJ/Bl6 mice and were bred and maintained in the Hubrecht Institute Animal Facility. All mouse experimentation was approved by the Animal Experimentation Committee (DEC) from the Koninklijke Nederlandse Akademie van Wetenschappen (KNAW) and complied with existing European Union legislation and local standards.

#### Mouse bone marrow

Male 13-week-old C57BL/6 mice were used to extract bone marrow cells. Femurs and tibia were extracted, the bone ends were cut away to access the bone marrow, which was flushed out using a 22G syringe with HBSS (– calcium, – magnesium, – phenol red, Gibco, catalog no. 14175053) supplemented with Pen-Strep and 1% fetal calf serum. The bone marrow was dissociated and debris removed by passing through a 70 μm cell strainer (Corning, catalog no. 431751). Cells were washed with 25 ml supplemented HBSS before depleting the sample of unnucleated cells using IOTest 3 Lysing solution (Beckman Coulter) following the providerʼs instructions. Cells were washed an additional two times with PBS before processing them by the sortChIC protocol for histone modifications. For whole bone marrow experiments (that is, not enriched for specific cell types), we processed cells using the sortChIC protocol for unfixed cells (without ethanol fixation). For the ground truth experiment with sorted cell types, we processed cells using the sortChIC protocol for ethanol-fixed cells. For ethanol fixation, cells were resuspended in 70% ethanol and fixed for 1 h at –20 °C. Afterwards cells were resuspended in Storage buffer (42.5 ml H_2_O RNAse free, 1 ml 1 M HEPES pH 7.5 (Invitrogen), 1.5 ml 5 M NaCl, 3.6 μl spermidine (Sigma Aldrich, catalog no. S2626-5G), protease inhibitor (Sigma Aldrich, catalog no. 5056489001), 200 μl 0.5 M EDTA, 5 μl dimethylsulfoxide) and frozen at –80^∘^C, before processing by the sortChIC protocol.

#### Mouse organogenesis

No randomization or blinding was performed. Sex of embryos was not known at the time of collection. Four to five embryos were pooled for each reported timepoint (E9.5, E10.5, E11.5) before single-cell isolation. Pooled embryos were dissociated in TrypleE for 10 min at room temperature. Undigested portions were physically removed and the remainder filtered through a 30 μm filter before the single-cell suspension was split into three samples for each timepoint and each scChIX-seq experiment. Per timepoint, two single-cell samples were used each for a single antibody incubation (H3K36me3 or H3K9me3) and one sample for the double antibody incubation (H3K36me3 + H3K9me3). Antibody incubation was performed as described in the scChIX-seq protocol before single-cell capture using flow cytometry. A DNA library was prepared for each sample using the sortChIC protocol for unfixed cells.

#### In vitro macrophage differentiation

For in vitro differentiation of bone marrow-derived macrophages, bone marrow was collected aseptically by flushing tibia and femurs from euthanized wild-type male C57BL/6 mice with sterile RPMI and 10% FCS through a 70 μm cell strainer (Corning). To enrich for stem and progenitor cells, lineage marker-positive (Lin^+^) cells were depleted by magnetic-activated cell sorting using a mouse Lineage Cell Depletion kit (Miltenyi Biotec). Lin^–^ cells were cultured on nontissue-culture-treated plates (Corning) for 7 days in RPMI medium supplemented with 10% FCS, 100 IU ml^–1^ penicillin, 100 mg ml^–1^ streptomycin and 10 ng ml^–1^ recombinant murine MCSF (Peprotech). Medium was refreshed after 3 days. Every 24 h, suspension cells were collected and adherent cells were harvested by incubating 10 min in 2 mM EDTA/0.5% BSA in PBS. Suspension and adherent cells were combined and stained with CellTrace fluorescent labels (Thermo Fisher), according to manufacturer’s instructions. Briefly, cells were pelleted and resuspended in 37 °C PBS containing fluorescent dyes (working concentrations CellTrace CSFE (CTC): 2.5 μM; CellTrace Yellow (CTY): 2.5 μM; CellTrace Far Red (CTFR): 0.5 μM) at a concentration of 1,000,000 cells ml^–1^. Cells were incubated at 37 °C protected from light for 20 min. Staining reactions were stopped by adding two volumes of RPMI/10% FCS and incubating for 5 min at room temperature, protected from light, after which cells were washed twice in PBS. The following combinations of labels were used: unstained (day 0), CTC (day 1), CTY (day 2), CTFR (day 3), CTC + CTY (day 4), CTC + CTFR (day 5), CTY + CTFR (day 6) and CTC + CTY + CTFR (day 7). After harvesting and staining, cells were fixed in 70% ethanol for 1 h and stored for later by the sortChIC protocol for fixed cells.

### Cell preparation without ethanol fixation for sortChIC experiments

Cells from whole bone marrow (H3K4me1+H3K27me3) and mouse embryos (H3K36me3+H3K9me3) were processed using the sortChIC method without ethanol fixation. Cells were processed in 0.5 ml protein low-binding tubes. Following steps were performed on ice. Cells were resuspended in 500 μl wash buffer (47.5 ml H_2_O RNAse free, 1 ml 1 M HEPES pH 7.5 (Invitrogen), 1.5 ml 5M NaCl, 3.6 μl pure spermidine solution (Sigma Aldrich)). Cells were pelleted at 600*g* for 3 min and resuspended in 400 μl wash buffer 1 (wash buffer with 0.05% saponin (Sigma Aldrich), protease inhibitor cocktail (Sigma Aldrich), 4 μl 0.5 M EDTA) containing the primary antibody (1:100 dilution for the antibody, saponin has to be prepared fresh every time as a 10% solution in PBS). Cells were incubated overnight at 4 °C on a roller, before being washed once with 500 μl wash buffer 2 (wash buffer with 0.05% saponin, protease inhibitor). Afterwards cells were resuspended in 500 μl wash buffer 2 containing Protein A-Micrococcal Nuclease (pA-MNase) (3 ng ml^–1^) and incubated for 1 h at 4 °C on a roller.

Finally, cells were washed an additional two times with 500 μl wash buffer 2 before passing it through a 70 μm cell strainer (Corning, catalog no. 431751) and sorting G1 cells based on Hoechst staining on a BD Influx FACS machine into 384-well plates containing 50 nl wash buffer 3 (wash buffer containing 0.05% saponin) and 5 μl sterile filtered mineral oil (Sigma Aldrich) per well. Small volumes were distributed using a Nanodrop II system (Innovadyme).

### Cell preparation with ethanol fixation and surface antibody incubation for sortChIC experiments

Cells from sorted bone marrow (H3K27me3+H3K9me3) and macrophage in vitro differentiation (H3K4me1+H3K36me3) were processed using the ethanol fixation protocol. Sorted bone marrow cells were also incubated with surface antibody to enrich for known cell types. For the ethanol-fixed cells the above described sortChIC protocol was adapted. Wash buffers were used as described above, except that 0.05% saponin was exchanged for 0.05% Tween. Ethanol-fixed cells were thawed on ice. Cells were spun at 400*g* for 5 min and washed once with 400 μl wash buffer 1. Cells were spun again at 400*g* and resuspended in 400 μl wash buffer 1. Cell suspension was split into three samples each having a volume of 400 μl and incubated with one or two antibodies (1:100 dilution for the antibody) overnight on a roller at 4 °C. The next day, cells were spun at 400*g*, washed once with 400 μl wash buffer 2 and resuspended in 500 μl wash buffer 2 containing pA-MNase (3 ng ml^–1^) and incubated for 1 h on a rotator at 4 °C. Next, cells were spun at 400*g* and resuspended in 400 μl wash buffer 2 (with addition of 5% blocking rat serum). To sort for defined cell types in the ground truth bone marrow experiment, surface antibodies were added according to these concentrations and were incubated for 30 min on ice:

$$\begin{array}{l}\begin{array}{ll}{{\mbox{antibody}}}\,&\,{{\mbox{info}}}\\ {{\mbox{GR1}}}\,&\,{{\mbox{A647, anti-mouse Ly-6G/Ly-6C (Gr-1) Antibody,}}}\\ & {\mbox{clone: RB6-8C5}}\\ {{\mbox{NK1}}}\,&\,{{\mbox{A488, anti-mouse NK-1.1 Antibody, clone: PK136}}}\\ {{\mbox{CD19}}}\,&\,{{\mbox{BV421, anti-mouse CD19 Antibody, clone: 6D5}}}\end{array}\\\begin{array}{l}{{\mbox{working concentration}}}\\1:8,000\\1:400\\1:200\end{array}\end{array}$$

BD FAC software v.1.2.0.142 was used to collect data from the FACS machine during cell sorting; see Supplemental Fig. 1 for the gating strategy.

Finally, samples were washed once with 500 μl wash buffer 2 before passing them through a 70 μm cell strainer (Corning, catalog no. 431751) and sorting on a BD Influx FACS machine, with surface antibody specific gating, into 384-well plates containing 50 nl wash buffer 3 (wash buffer containing 0.05% Tween) and 5 μl sterile filtered mineral oil (Sigma Aldrich) per well. Small volumes were distributed using a Nanodrop II system (Innovadyme).

### MNase activation for sortChIC experiments

Targeted fragmentation was started by the addition of 5 μl wash buffer 2 containing 4 mM CaCl_2_. For digestion, plates were incubated for 30 min in a PCR machine set at 4 °C. Afterwards the reaction was stopped by adding 100 nl of a stop solution containing 40 mM EGTA, 1.5% NP40, and 10 nl 2 mg ml^−1^ proteinase K. Plates were incubated in a PCR machine for further 20 min at 4 °C, before chromatin was released and pA-MNase permanently destroyed by proteinase K digestion at 65 °C for 6 h followed by 80 °C for 20 min to heat inactivate proteinase K. Afterwards plates were stored at –80 °C until further processing.

### Library preparation for sortChIC experiments

DNA fragments were blunt ended by adding 150 nl end repair mix per well and incubating for 30 min at 37 °C followed by 20 min at 75 °C for enzyme inactivation. End repair mix per well: Klenow large (NEB, catalog no. M0210L) 2.5 nl, T4 PNK (NEB, catalog no. M0201L) 2.5 nl, dNTPs 10 mM 6 nl, ATP 100 mM 3.5 nl, MgCl_2_ 25 mM 10 nl, PEG8000 50% 7.5 nl, PNK buffer 10× (NEB, catalog no. B0201S) 35 nl, BSA 20 ng 1.8 nl, nuclease-free water 81.3 nl.

Blunt fragments were subsequently A-tailed by adding 150 nl per well of A-tailing mix and incubated for 15 min at 72 °C. Through the strong preference of AmpliTaq 360 to incorporate dATP as a single base overhang even in the presence of other nucleotides, a general dNTP removal was not necessary. A-tailing mix per well: AmpliTaq 360 (Thermo Fisher Scientific, catalog no. 4398828) 1 nl, dATPs 100 mM 1 nl, KCl 1 M 25 nl, PEG8000 50% 7.5 nl, BSA 20 ng 0.8 nl, nuclease-free water 114.8 nl.

Fragments were ligated to T-tail containing forked adapters containing a T7 polymerase binding site for in vitro transcription (IVT)-based amplification.

Top strand: 5′-GGTGATGCCGGTAATACGACTCACTATAGGGAGTTCTACAGTCCGACGATCNNNACACACTAT-3′

Bottom strand: 5′-TAGTGTGTNNNGATCGTCGGACTGTAGAACTCCCTATAGTGAGTCGTATTACCGGCGAGCTT-3′

The three random nucleotides (NNN) were the unique molecular identifier used for read deduplication and the eight bases afterwards represent the cell barcodes, which were different for each of the 384 wells. For a full list of adapters and the cell barcodes for each well, see the excel sheet in Supplemental Table 1. Cell barcodes for each 384-well plates are also found as a text file in the scChIX-seq Github repository: (https://github.com/jakeyeung/scChIX/blob/main/inst/extdata/cellbarcodes_384_NLA_annotated.bc).

For ligation, 50 nl of 5 μM adapter in 50 mM Tris pH 7 was added to each well with a Mosquito HTS (ttp labtech). After centrifugation, 150 nl of ligation mix was added before incubating plates for 20 min at 4 °C, followed by 16 h at 16 °C for ligation and 10 min at 65 °C to inactivate ligase. Adapter ligation mix per well: T4 ligase (400,000 U ml^–1^, NEB, catalog no. M0202L) 25 nl, MgCl_2_ 1 M 3.5 nl, Tris 1 M pH 7.5 10.5 nl, DTT 0.1 M 52.5 nl, ATP 100 mM 3.5 nl, PEG8000 50% 10 nl, BSA 20 ng 1 nl, nuclease-free water 44 nl.

Before pooling, 1 μl nuclease-free water was added to each well to minimize material loss. Ligation products were pooled by centrifugation into oil coated VBLOK200 Reservoir (ClickBio) at 500*g* for 2 min and the liquid face was transferred into 1.5 ml Eppendorf tubes and then purified by centrifugation at 13,000*g* for 1 min and transferred into a fresh tube twice. DNA fragments were purified using Ampure XP beads (Beckman Coulter, prediluted one in eight in bead binding buffer: 1 M NaCl, 20% PEG8000, 20 mM Tris pH 8, 1 mM EDTA) at a bead to sample ratio of 0.8. After 15 min incubation at room temperature, beads were washed twice with 1 ml 80% ethanol resuspending the beads during the first wash and resuspended in 20 μl nuclease-free water. After 2 min elution, the supernatant was transferred into a fresh 0.5 ml tube. A second cleanup was performed adding 26 μl undiluted Ampure XP beads and the beads were resuspended in 8 μl nuclease-free water. The cleaned DNA was then linear amplified by IVT by adding 12 μl of MEGAscript T7 Transcription Kit (Fisher Scientific, catalog no. AMB13345) for 12 h at 37 °C. Template DNA was removed by addition of 2 μl^–1^ TurboDNAse (IVT kit) and incubation for 15 min at 37 °C. The RNA produced was further purified using RNA Clean XP beads (Beckman Coulter) at a beads to sample ratio of 0.8 and samples were resuspended in 22 μl of nuclease-free water. RNA was fragmented by mixing in 4.4 μl fragmentation buffer (200 mM Tris-acetate pH 8.1, 500 mM KOAc, 150 mM MgOAc) and incubation for 2 min at 94 °C. Fragmentation was stopped by transferring samples to ice, adding 2.64 μl 0.5 M EDTA and another bead cleanup; samples were resuspended in 12 μl nuclease-free water.

RNA (5 μl) was primed for reverse transcription by adding 0.5 μl 10 mM dNTPs and 1 μl 20 mM randomhexamerRT primer (5′-GCCTTGGCACCCGAGAATTCCANNNNNN-3′) and hybridizing it by incubation at 65 °C for 5 min followed by direct cool down on ice. Reverse transcription was performed by further addition of 2 μl first strand buffer (part of Invitrogen kit, catalog no. 18064014), 1 μl 0.1 M DTT, 0.5 μl RNAseOUT (Invitrogen, catalog no. LS10777019) and 0.5 μl SuperscriptII (Invitrogen, catalog no. 18064014) and incubating the mixture at 25 °C for 10 min followed by 1 h at 42 °C. Single-stranded DNA was purified through incubation with 0.5 μl RNAseA (Thermo Fisher, catalog no. EN0531) and incubation for 30 min at 37 °C.

A final PCR amplification to add the Illumina small RNA barcodes and handles was performed by adding 25 μl of NEBNext Ultra II Q5 Master Mix (NEB, catalog no. M0492L), 11 μl nuclease-free water and 2 μl of 10 μM RP1 and RPIx primers.

### PCR protocol for sortChIC experiments

Activation for 30 s at 98 °C, 8–12 cycles (depending on starting material), 10 s at 98 °C, 30 s at 60 °C, 30 s at 72 °C, final amplification 10 min at 72 °C.

PCR products were cleaned by two consecutive DNA bead clean-ups with a bead to sample ratio of 0.8. Final product was eluted in 7 μl nuclease-free water. The abundance and quality of the final library were assessed by QUBIT and bioanalyzer.

### Data processing

All DNA libraries were sequenced on a Illumina NextSeq500 with 2 × 75 bp. We ran the raw fastq files through the Single-Cell MultiOmics (SCMO) workflow (github.com/BuysDB/SingleCellMultiOmics^52^). The workflow comprises of six steps.

(1) Demultiplex raw fastq files using demux.py (SCMO). (2) Trim fastq files by removing adapters using cutadapt (v.3.5). (3) Map trimmed fastq files using bwa (v.0.7.17-r1188). (4) Tag bam files with cell barcode information, using bamtagmultiome.py (SCMO). (5) Generate count tables using bamToCountTable.py (SCMO). (6) Run dimensionality reduction of count matrices using run_LDA_model.R. See an example of the pipeline in the scChIX-seq Github repository^53^.

### Unmixing scChIX-seq signal

Single-cell epigenomics techniques (for example, sortChIC, CUT&RUN and CUT&TAG) generate a vector of counts indicating the number of cut fragments that map in each genomic region for each cell. We model the vector of counts from a double-incubated cell $\overrightarrow{y}$ as a linear combination of two multinomial distributions: one coming from a cluster *c* of histone modification 1, parameterized by ${\overrightarrow{p}}_{c}$, the other from another cluster *d* of histone modification 2 ${\overrightarrow{q}}_{d}$. The log-likelihood for a linear combination of two multinomials is:

1 $${{{{\rm{L}}}}}_{(c,d)}=\log (P\left(\overrightarrow{y}| {\overrightarrow{p}}_{c},{\overrightarrow{q}}_{d},w\right))\propto \mathop{\sum }\limits_{g=1}^{G}{y}_{g}\log \left(w{p}_{c,g}+\left(1-w\right){q}_{d,g}\right).$$

$\overrightarrow{y}$ is the number of cuts across the genome for a double-incubated cell. *p*_*c*,*g*_ and *q*_*d*,*g*_ are cluster-specific probabilities indicating the likelihood that a cut fragment maps to region *g* in histone modifications 1 and 2, respectively. *w* is the mixing fraction of histone modification 1 in the double-incubated cell, which we estimate by maximizing the log-likelihood given $\overrightarrow{y}$, ${\overrightarrow{p}}_{c}$ and ${\overrightarrow{q}}_{d}$.

Applying single-cell techniques to complex tissues generates data with many clusters. Therefore, given a double-incubated cell, we do not know which pair of clusters (*c*,*d*) were combined to generate the observed counts. We therefore calculate the log-likelihood for all possible pairs of clusters learned from the training data and then select the cluster pair with the highest probability for each cell.

Cluster-specific probabilities ${\overrightarrow{p}}_{c}$ and ${\overrightarrow{q}}_{d}$ are learned by applying LDA (with k = 30 topics) using the topicmodels R package^54^ to the training data (that is, single-incubated cells), which are count matrices.

After assigning each cell to the most probable cluster pair $(\hat{c},\hat{d})$, we assign *y*_i,j_, the jth read mapped to region *g* in cell *i*, to histone mark 1 with probability *P*_i,j_:

2 $${P}_{\mathrm{i,j}}=\frac{w{p}_{\hat{c},g}}{w{p}_{\hat{c},g}+\left(1-w\right){q}_{\hat{d},g}}.$$

This assignment generates a pair of vectors ${\overrightarrow{y}}_{1,i}$ and ${\overrightarrow{y}}_{2,i}$ that are linked because they both come from cell *i*. Unmixed counts ${\overrightarrow{y}}_{1,i}$ and ${\overrightarrow{y}}_{2,i}$ are then projected back onto the space inferred from training data of histone modification 1 and 2, respectively. The links between histone modification 1 and 2 are used to transfer labels and create linked UMAPs between the two histone modifications.

### Latent Dirichlet allocation

LDA is a probabilistic matrix decomposition model that is useful when the input data is a matrix of counts. LDA uses hierarchical multinomial models to estimate the relative frequencies of cuts in each genomic region in single cells.

To generate the genomic location of the jth read for cell i:

Choose a topic *z*_i,j_ by sampling from the cell-specific distribution of topics:

$$\begin{array}{r}{\overrightarrow{U}}_{\mathrm{i}} \sim \,{{{\rm{Dirichlet}}}}\,(\alpha )\\ {z}_{\mathrm{i,j}} \sim \,{{{\rm{Multinomial}}}}\,({\overrightarrow{U}}_{i},1)\end{array}$$

Choose genomic region *w*_i,j_ by sampling from the topic-specific distribution of genomic regions:

$$\begin{array}{r}{\overrightarrow{V}}_{\mathrm{k}} \sim \,{{{\rm{Dirichlet}}}}\,(\delta )\\ {w}_{\mathrm{i,j}} \sim \,{{{\rm{Multinomial}}}}\,({\overrightarrow{V}}_{{z}_{\mathrm{i,j}}},1)\end{array}$$

The Dirichlet distributions are priors to prevent overfitting when there are few cuts in the region. We used the LDA model implemented by the topicmodels R package, using the Gibbs sampling implementation with hyperparameters *α* = 1.67, *δ* = 0.1, where K is the number of topics^23^.

We estimate ${\overrightarrow{p}}_{c}$ and ${\overrightarrow{q}}_{d}$ for each cluster in histone modification 1 $\{{\overrightarrow{p}}_{1},{\overrightarrow{p}}_{2},...,{\overrightarrow{p}}_{C}\}$ and modification 2 $\{{\overrightarrow{q}}_{1},{\overrightarrow{q}}_{2},...,{\overrightarrow{q}}_{D}\}$ by averaging the estimated probabilities across cells assigned to each cluster for each gene *g*:

$$p_{g,c}=\frac{1}{\vert C \vert}\mathop{\sum }\limits_{\mathrm{i}\in C}\mathop{\sum }\limits_{\mathrm{k}=1}^{K}{V}_{\mathrm{g,k}}{U}_{\mathrm{k,i}}$$

where *C* is the set of cells that belong to cluster *c*.

### Simulation of single- and double-incubated histone modification data

To simulate multimodal single-cell histone modification data with varying degrees of overlap, we extended simATAC^55^ to allow generating cell-type profiles from histone modifications of varying mutually exclusive relationships.

For each cell type, we first run simATAC to generate sparse count data of 10,000 loci across 750 cells partitioned into three technical replicates of 250 cells each. The high-dimensional count data are sparse. Counts from each locus are generated according to a Poisson likelihood with locus-specific means (*λ*) matching real single-cell ATAC-seq from K562 cells (GSE99172).

In our 750 cells, cells 1–250 represent single-incubated cells from mark 1; cells 251–500 from mark 2; cells 501–750 from double-incubated cells. Cells from mark 1 have counts generated from locus-specific means *λ*. Cells from mark 2 also have counts generated from *λ*, but we swap the top *x*% of bins with highest *λ* with bins with lowest *λ*, allowing precisely defined sets of mutually exclusive and overlapping bins. We use *x* = 1%, 50% and 99% to benchmark our method from mostly overlapping (that is *x* = 1%) to mostly mutually exclusive (that is *x* = 99%) Cells from mark 3 are generated by adding counts generated from mark 1 and mark 2 to simulate double-incubated cells.

To generate cell-type-specific profiles, we repeat the above with a cell-type-specific random seed and shuffle the order of the bins. This generates count data where *λ* is cell-type specific, but the distribution of *λ* are preserved genome-wide.

### Estimating the top cluster-specific bins

We use the LDA matrix factorization to identify the top cluster-specific bins in the data. We rank the bin loadings for each cell type and take the top 150 (whole bone marrow) or 250 (mouse organogenesis) bins with the largest loadings.

### Inferring pseudotime in differentiation data

To analyze the macrophage differentiation data, we first removed erythroblasts, plasmacytoid dendritic cells, and innate lymphocyte cells from the data, which were concentrated at day 0 and not considered to be part of the macrophage differentiation trajectory. We then ran LDA (k = 30 topics) and performed principal component analysis (PCA) on the LDA outputs, which retrieves the principal components that explain the largest amount of variance after denoising the data. We used the first principal component for H3K4me1 and H3K36me3 to define pseudotime, which we found correlates with the day along the timecourse.

### Unmixing scChIX-seq signal from continuous pseudotime

To apply scChIX-seq on continuous pseudotime, we modify the log-likelihood (equation (1)) to account for a continuous variable:

3 $${{{\rm{L}}}}\left({t}_{1},{t}_{2}\right)=\log \left(P\left(\overrightarrow{y}| \overrightarrow{p}\left({t}_{1}\right),\overrightarrow{q}\left({t}_{2}\right),w\right)\right)\propto \mathop{\sum }\limits_{g=1}^{G}{y}_{g}\log \left(w{p}_{g}\left({t}_{1}\right)+\left(1-w\right){q}_{g}\left({t}_{2}\right)\right)$$

where *t*_1_ ∈ [0, 1] is pseudotime from histone modification 1 and *t*_2_ ∈ [0, 1] is pseudotime from modification 2.

To estimate pseudotime, we ran LDA to denoise the count matrix, and then ran PCA to estimate largest principal components explaining the variance in the data. We took the first principal component as our pseudotime estimate for both marks, which captured the epigenomic changes over the 7-day timecourse.

${p}_{g}\left(t\right)$ is estimated by fitting the signal from histone modification 1 at genomic region *g* with a lowess curve along pseudotime. We estimate *q*_*g*_ analogously but using signal from histone modification 2.

To infer the pseudotime of histone modifications 1 and 2 simultaneously given a vector of counts from a double-incubated cell, we estimate *t*_1_ and *t*_2_ that minimizes the log-likelihood *L* from equation (3). We estimate the variance-covariance matrix of *t*_1_ and *t*_2_ by the square root of the inverse of the Hessian matrix, which we use to calculate the standard errors.

Since the *t*_1_ and *t*_2_ are constrained between 0 and 1, we use the L-BFGS-B optimization algorithm implemented in R. Since estimates from a single cell can sometimes be noisy due to low counts, we sum the counts across the 25-nearest neighbors (estimated from the latent space inferred by LDA) for each double-incubated cell.

### Chromatin velocity during macrophage differentiation

We assume that dynamic genomic regions in H3K36me3 can be modeled using a first-order differential equation

4 $$\frac{d{K}_{36}\left(t\right)}{dt}={K}_{4}\left(t\right)-\gamma {K}_{36}\left(t\right).$$

We estimate the time constant *γ* for each genomic region by fitting an exponential relaxation function across pseudotime

5 $${K}_{36}\left(t\right)={y}_{0}+A\left(1-{e}^{-\gamma t}\right),$$

where *y*_0_ is the signal at *t* = 0 and *A* is the predicted H3K36me3 levels at steady state. Fitting the *γ* directly from the pseudotime allows us to leverage signal from both single- and deconvolved cells.

To predict future values of H3K36me3 levels for each cell at each genomic region, we use the Euler method and plug in the estimated *γ*, H3K4me1 levels at time *t* and time step *h* of 0.02 pseudotime units:

6 $${K}_{36}\left(t+1\right)={K}_{36}\left(t\right)+h\left({K}_{4}\left(t\right)-\gamma {K}_{36}\left(t\right)\right).$$

Finally, we project the single- and double-incubated H3K36me3 signal onto the first two principal components and project the predicted future values onto the PCA. We use the velocity grid flow visualization as implemented in velocyto^56^ to visualize the velocity vectors on the PCA space.

### Comparison with multi-CUT&TAG

Raw fastq files (R1, R2 and R3) from the single-cell experiments were downloaded from Gene Expression Omnibus accession number GSE171554. The first 42 bases of the reads in R1 and R2 were trimmed to remove the barcodes and the bases common to all Tn5 adapter sequences. The 16-base cell barcodes in R3 were added to the fastq headers of R1 and R2. The trimmed and cell-barcoded R1 and R2 reads were then aligned to the mm10 mouse genome using Burrows-Wheeler aligner (bwa v.0.7.17-r1188). Fragments that start at same location and have the same cell barcode were considered duplicates and discarded. Cells with more than 100 fragments with MAPQ scores in R1 greater than or equal to 40 were kept for comparison with scChIX-seq.

### Reporting summary

Further information on research design is available in the Nature Portfolio Reporting Summary linked to this article.

## Online content

Any methods, additional references, Nature Portfolio reporting summaries, source data, extended data, supplementary information, acknowledgements, peer review information; details of author contributions and competing interests; and statements of data and code availability are available at 10.1038/s41587-022-01560-3.

### Supplementary information

Supplementary information Table of contents, Supplementary Fig. 1 and captions.

Reporting Summary

Supplementary Table 1 Full adapter sequences and cell barcodes.

## Data Availability

The data discussed in this publication have been deposited in NCBI’s Gene Expression Omnibus and are accessible through Gene Expression Omnibus Series accession number GSE155280 (ref. ^57^).

## Code availability

We developed the SingleCellMultiOmics package, in which there are modules used for processing sortChIC data (https://github.com/BuysDB/SingleCellMultiOmics)^52^, and an R package that implements scChIX-seq and contains snakemake workflows for processing data and example notebooks for downstream analyses (https://github.com/jakeyeung/scChIX)^53^.
