# Supplementary material for: scChIX-seq infers dynamic relationships between histone modifications in single cells
Source: Nat Biotechnol. 2023 Jan 2;41(6):813–23. doi: 10.1038/s41587-022-01560-3 (PMC10264247; doi:10.1038/s41587-022-01560-3)
Supplement: Supplementary file 1 — Table of contents, Supplementary Fig. 1 and captions. [file 41587_2022_1560_MOESM1_ESM.pdf]

nature biotechnology

Article

https://doi.org/10.1038/s41587-022-01560-3

scChIX-seq infers dynamic relationships between histone modifications in single cells

In the format provided by the authors and unedited

Table of Contents

SUPPLEMENTAL FIGURE 1 2

Supplementary Figure 1

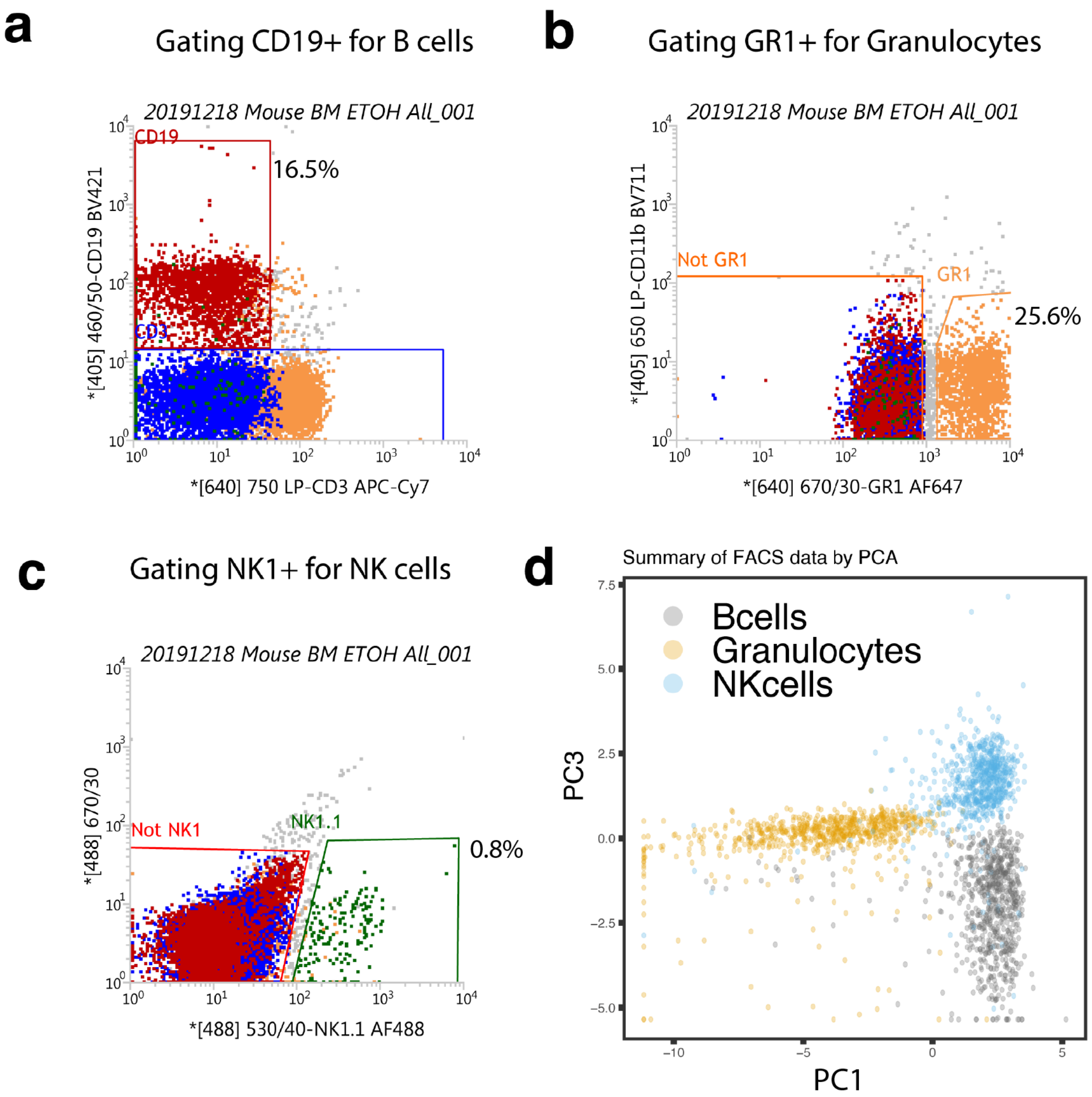

Supplementary Figure 1: fluorescence-activated cell sorting (FACS) gating strategy. **(a)** Gating strategy to purify for B cells by staining for CD19+. **(b)** Gating strategy to purify for granulocytes by staining for GR1+. **(c)** Gating strategy to purify for NK cells by staining for NK1+. **(d)** Summary of the high-dimensional FACS information projected onto principal components 1 and 3, showing separation of the three cell types in FACS space.
