# Supplementary material for: scChIX-seq infers dynamic relationships between histone modifications in single cells
Source: Nat Biotechnol. 2023 Jan 2;41(6):813–23. doi: 10.1038/s41587-022-01560-3 (PMC10264247; doi:10.1038/s41587-022-01560-3)
Supplement: Supplementary file 2 — Reporting Summary [file 41587_2022_1560_MOESM2_ESM.pdf]

nature research

Corresponding author(s): Alexander van Oudenaarden

Last updated by author(s): Sep 2, 2022

Reporting Summary

Nature Research wishes to improve the reproducibility of the work that we publish. This form provides structure for consistency and transparency in reporting. For further information on Nature Research policies, see our Editorial Policies and the Editorial Policy Checklist.

Statistics

For all statistical analyses, confirm that the following items are present in the figure legend, table legend, main text, or Methods section.

n/a	Confirmed	
☐	☒	The exact sample size (*n*) for each experimental group/condition, given as a discrete number and unit of measurement
☐	☒	A statement on whether measurements were taken from distinct samples or whether the same sample was measured repeatedly
☒	☐	The statistical test(s) used AND whether they are one- or two-sided<br>*Only common tests should be described solely by name; describe more complex techniques in the Methods section.*
☐	☒	A description of all covariates tested
☐	☒	A description of any assumptions or corrections, such as tests of normality and adjustment for multiple comparisons
☐	☒	A full description of the statistical parameters including central tendency (e.g. means) or other basic estimates (e.g. regression coefficient) AND variation (e.g. standard deviation) or associated estimates of uncertainty (e.g. confidence intervals)
☒	☐	For null hypothesis testing, the test statistic (e.g. *F*, *t*, *r*) with confidence intervals, effect sizes, degrees of freedom and *P* value noted<br>*Give P values as exact values whenever suitable.*
☐	☒	For Bayesian analysis, information on the choice of priors and Markov chain Monte Carlo settings
☒	☐	For hierarchical and complex designs, identification of the appropriate level for tests and full reporting of outcomes
☐	☒	Estimates of effect sizes (e.g. Cohen's *d*, Pearson's *r*), indicating how they were calculated

Our web collection on statistics for biologists contains articles on many of the points above.

Software and code

Policy information about availability of computer code

Data collection	BD FACS™ Sortware 1.2.0.142 was used to collect data from the FACS machine during cell sorting.
Data analysis	Python package for processing fastq files into bams: https://github.com/BuysDB/SingleCellMultiOmics<br>R package associated with deconvolving the signal: https://github.com/jakeyeung/scChIX

For manuscripts utilizing custom algorithms or software that are central to the research but not yet described in published literature, software must be made available to editors and reviewers. We strongly encourage code deposition in a community repository (e.g. GitHub). See the Nature Research guidelines for submitting code & software for further information.

Data

Policy information about availability of data

All manuscripts must include a data availability statement. This statement should provide the following information, where applicable:

- Accession codes, unique identifiers, or web links for publicly available datasets
- A list of figures that have associated raw data
- A description of any restrictions on data availability

Data has been uploaded to Gene Expresion Omnibus (GEO) accession number: GSE155280.

Field-specific reporting

Please select the one below that is the best fit for your research. If you are not sure, read the appropriate sections before making your selection.

☒ Life sciences ☐ Behavioural & social sciences ☐ Ecological, evolutionary & environmental sciences

For a reference copy of the document with all sections, see nature.com/documents/nr-reporting-summary-flat.pdf

Life sciences study design

All studies must disclose on these points even when the disclosure is negative.

Sample size

No sample-size calculation was performed. The number of plates we used was based on our estimates of the number of different cell types we expected to see. For the ground truth experiment, we expected only a few cell types, and therefore used 9 plates (i.e. 3 plates per antibody condition). For organogenesis where we expected more cell types, we used 33 plates (13 plates for H3K36me3, 10 plates for H3K9me3, and 10 plates for dual-incubation). For macrophage in vitro differentiation, we used 8 plates per antibody condition.

Data exclusions

Cells that did not pass quality controls were excluded from the analysis. We removed all cells that had fewer than 50 percent of reads starting with a TA sequence (removed for low MNase specificity). In bone marrow H3K27me3 and H3K9me3 samples, we further removed cells that had fewer than 1000 unique cuts. In bone marrow H3K4me1, we removed cells that had fewer than 500 unique cuts. For mouse organogenesis, we removed cells that had fewer than 1000 unique cuts. For macrophage differentiation, we removed cells that had fewer than 3000 unique cuts.

Replication

We performed experiments across multiple plates and found the results across these technical replicates to be reproducible. When projecting cells across technical replicates onto a low-dimensional manifold, we did not observe effects coming from differences in technical replicates.

Randomization

We used blocking in the experimental plate design to reduce unexplained variability, within each block the cells were randomly assigned onto the plates. In the ground truth experiment, we minimized effects across plates by sorting different cell types onto the same plate. The location of each cell was not randomly assigned to the well on the plate. For macrophage in vitro differentiation, samples were collected over 7 days, but FACS sorting was done onto plates to pool 7 days of samples evenly onto the plates to reduce batch effect. For organogenesis experiment, different plates corresponded to different stages of development.

Blinding

No blinding was done because the experiments did not involve conditions that would induce a bias from the experimentalist.

Reporting for specific materials, systems and methods

We require information from authors about some types of materials, experimental systems and methods used in many studies. Here, indicate whether each material, system or method listed is relevant to your study. If you are not sure if a list item applies to your research, read the appropriate section before selecting a response.

Materials & experimental systems

n/a	Involved in the study
☐	☒ Antibodies
☒	☐ Eukaryotic cell lines
☒	☐ Palaeontology and archaeology
☐	☒ Animals and other organisms
☒	☐ Human research participants
☒	☐ Clinical data
☒	☐ Dual use research of concern

Methods

n/a	Involved in the study
☐	☒ ChIP-seq
☐	☒ Flow cytometry
☒	☐ MRI-based neuroimaging

Antibodies

Antibodies used

H3K4me1: rabbit anti-mouse H3K4me1, polyclonal, Ab8895, Lot: GR3206285-1, Abcam
H3K27me3: rabbit anti-mouse H3K27me3, monoclonal, Identifier: 9733S, NEB
H3K9me3: rabbit anti-mouse H3K9me3, polyclonal, Ab8898, Lot: GR3217826-1, Abcam
H3K36me3: rabbit anti-mouse H3K36me3, monoclonal, clone: RM155, Merck
GR1: A647, rat anti-mouse Ly-6G/Ly-6C, monoclonal, clone: RB6-8C5, Lot: 108420, Biolegend
NK1: A488, rat anti-mouse anti NK-1.1, clone: PK136, Lot: 108717, Biolegend
CD19: BC421, rat anti-mouse CD19, clone: 6D5, Lot: 11537, Biolegend
Haematopoietic stem and progenitor enrichment pool: mix of biotinylated antibodies against CD5, CD11b, CD19, CD45R/B220, Ly6G/C(Gr-1), TER119, 7-4, part of #19856, Stemcell

Validation

We validated antibodies by performing sortChIC on K562 cells and confirmed that we reproduced the publicly available ChIP-seq

Validation	signals from the ENCODE project.

Animals and other organisms

Policy information about studies involving animals; ARRIVE guidelines recommended for reporting animal research

Laboratory animals	Male 13-week-old C57BL/6 mice were used to extract bone marrow cells. Embryos from E9.5, E10.5, and E11.5 were used to extract cells for mouse organogenesis study. Mice were kept in 12h:12h light:dark cycles in controlled ambient temperature and humidity, food and water ad libitum.
Wild animals	No wild animals were used in this study.
Field-collected samples	No field-collected samples were used in this study.
Ethics oversight	Experimental procedures were approved by the Dier Experimenten Commissie of the Royal Netherlands Academy of Arts and Sciences and performed according to the guidelines.

Note that full information on the approval of the study protocol must also be provided in the manuscript.

ChIP-seq

Data deposition

☒ Confirm that both raw and final processed data have been deposited in a public database such as GEO.

☒ Confirm that you have deposited or provided access to graph files (e.g. BED files) for the called peaks.

Data access links<br>*May remain private before publication.*	https://www.ncbi.nlm.nih.gov/geo/query/acc.cgi?acc=GSE155280<br><br>GEO Accession number: GSE155280
Files in database submission	bam<br>bigwigs<br>processed count tables
Genome browser session<br>(e.g. UCSC)	Bigwig files can be downloaded and directly viewed using IGV viewer

Methodology

Replicates	Validation experiments using ground truth cell types were performed across three technical plates. Experiments from whole bone marrow were performed across two technical plates.
Sequencing depth	In validation experiments, we sequenced to a mean depth of 48343 unique cut fragments per cell. In experiments from whole bone marrow, we sequenced to a mean depth of 9041 unique cut fragments per cell.
Antibodies	H3K4me1: rabbit anti-mouse H3K4me1, polyclonal, Ab8895, Lot: GR3206285-1, Abcam<br>H3K27me3: rabbit anti-mouse H3K27me3, monoclonal, Identifier: 9733S, NEB<br>H3K9me3: rabbit anti-mouse H3K9me3, polyclonal, Ab8898, Lot: GR3217826-1, Abcam<br>H3K36me3: rabbit anti-mouse H3K36me3, monoclonal, clone: RM155, Merck
Peak calling parameters	No peak calling was performed in this study.
Data quality	We removed all cells that had fewer than 50 percent of reads starting with a TA sequence (removed for low MNase specificity).<br>For ground truth bone marrow study: H3K27me3 and H3K9me3 samples, we further removed cells that had fewer than 1000 unique cuts.<br>For whole bone marrow study: H3K27me3 and H3K4me1 used cut off of 1000 and 500 unique cuts, respectively.<br>For mouse organogenesis, we removed cells that had fewer than 1000 unique cuts.<br>For macrophage differentiation, we removed cells that had fewer than 3000 unique cuts.
Software	Python package for processing fastq files into bams: https://github.com/BuysDB/SingleCellMultiOmics<br>R package associated with deconvolving the signal: https://github.com/jakeyeung/scChIX

Flow Cytometry

Plots

Confirm that:

☒ The axis labels state the marker and fluorochrome used (e.g. CD4-FITC).

☒ The axis scales are clearly visible. Include numbers along axes only for bottom left plot of group (a 'group' is an analysis of identical markers).

☒ All plots are contour plots with outliers or pseudocolor plots.

☒ A numerical value for number of cells or percentage (with statistics) is provided.

Methodology

Sample preparation	Ethanol fixed cells were thawed on ice. Cells were spun at 400 g for 5 minutes and washed once with 400 microlitre Wash Buffer 1 (47.5 ml H2O RNAse free, 1 ml 1 M HEPES pH 7.5 (Invitrogen), 1.5 ml 5M NaCl, 3:6 µl pure spermidine solution (Sigma Aldrich), 0:05% saponin). Cells were spun again at 400 g and resuspended in 400 microlitre Wash Buffer 1. Cell suspension was split into 3 samples each having a volume of 400 microlitre and incubated with one or two antibodies (1:100 dilution for H3K27me3, H3K9me3 and H3K27me3+H3K9me3) overnight on a roller at 4 degrees Celsius. The next day cells were spun at 400 g, washed once with 400 microlitre Wash Buffer 2 and resuspended in 500 microlitre Wash Buffer 2 containing pA-MNase (3 ng/mL) and incubated for 1 hour on a rotator at 4 degrees Celsius.<br>Next, cells were spun at 400 g and resuspended in 400 microliter Wash Buffer 2 (with addition of 5% blocking rat serum). Surface antibodies were added according to these concentrations and were incubated for 30 minutes on ice:<br><br>GR1 & A647, anti-mouse Ly-6G/Ly-6C (Gr-1) Antibody, clone: RB6-8C5 & 1:8000<br>NK1 & A488, anti-mouse NK-1.1 Antibody, clone: PK136 & 1:400<br>CD19 & BV421, anti-mouse CD19 Antibody, clone: 6D5 & 1:200<br><br>Finally, samples were washed once with 500 microlitre Wash Buffer 2 before passing them through a 70 micron cell strainer (Corning, 431751) and sorting on a BD Influx FACS machine, with surface antibody specific gating, into 384 well plates containing 50 nanoliter Wash buffer 3 (Wash buffer containing 0.05 % Tween) and 5 microlitre sterile filtered mineral oil (Sigma Aldrich) per well. Small volumes were distributed using a Nanodrop II system (Innovadyme).
Instrument	BD Influx System
Software	BD FACS™ Sortware 1.2.0.142
Cell population abundance	Purity of the sorted cell populations was assessed by performing scChIX and doing dimensionality reduction of chromatin levels across the genome in single cells.
Gating strategy	We used a forward scatter gate to remove debris (low FSC) and trigger pulse width to remove doublets (high trigger pulse width). We selected GR1+ cells as granulocytes, NK1.1+, GR1- cells as NK cells, and NK1.1-, GR1-, CD19+ cells are B cells.

☒ Tick this box to confirm that a figure exemplifying the gating strategy is provided in the Supplementary Information.
